# The composition of human vaginal microbiota transferred at birth affects offspring health in a mouse model

**DOI:** 10.1038/s41467-021-26634-9

**Published:** 2021-11-01

**Authors:** Eldin Jašarević, Elizabeth M. Hill, Patrick J. Kane, Lindsay Rutt, Trevonn Gyles, Lillian Folts, Kylie D. Rock, Christopher D. Howard, Kathleen E. Morrison, Jacques Ravel, Tracy L. Bale

**Affiliations:** 1 Center for Epigenetic Research in Child Health and Brain Development, University of Maryland, School of Medicine, Baltimore, MD 21201 USA; 2 Department of Pharmacology, University of Maryland School of Medicine, Baltimore, MD 21201 USA; 3 Department of Microbiology and Immunology, University of Maryland School of Medicine, Baltimore, MD 21201 USA; 4 Institute for Genome Sciences, University of Maryland School of Medicine, Baltimore, MD 21201 USA; 5 Department of Psychiatry, University of Maryland School of Medicine, Baltimore, MD 21201 USA; 6 Present Address: Department of Obstetrics, Gynecology and Reproductive Sciences, Magee-Womens Research Institute, University of Pittsburgh School of Medicine, Pittsburgh, PA 15213 USA

**Keywords:** Risk factors, Neutrophils, Microbiome, Systems analysis, Paediatric research

## Abstract

Newborns are colonized by maternal microbiota that is essential for offspring health and development. The composition of these pioneer communities exhibits individual differences, but the importance of this early-life heterogeneity to health outcomes is not understood. Here we validate a human microbiota-associated model in which fetal mice are cesarean delivered and gavaged with defined human vaginal microbial communities. This model replicates the inoculation that occurs during vaginal birth and reveals lasting effects on offspring metabolism, immunity, and the brain in a community-specific manner. This microbial effect is amplified by prior gestation in a maternal obesogenic or vaginal dysbiotic environment where placental and fetal ileum development are altered, and an augmented immune response increases rates of offspring mortality. Collectively, we describe a translationally relevant model to examine the defined role of specific human microbial communities on offspring health outcomes, and demonstrate that the prenatal environment dramatically shapes the postnatal response to inoculation.

## Abstract (web summary)

Exposure at birth to maternal microbiota has significant effects on offspring health and development. Here, the authors validate a model where inoculation of mice at birth with human vaginal microbiota produces significant effects on offspring health that are further amplified by an unhealthy prenatal environment.

## Introduction

The transmission of microbial communities from one generation to the next is a universal process in the animal kingdom, and exposure to this microbiota around birth is essential for optimal timing of development and health^1,2^. This intergenerational exchange appears to be specific and nonrandom, as highlighted by recent work demonstrating that the microbiota acquired from maternal body sites might be more ecologically adapted to colonize and persist in the infant gastrointestinal tract than microbiota from nonmaternal sources^3^. Preclinical studies also show associations between maternal microbial exposure and the recruitment of innate immune cells to the periphery, endotoxin tolerance, and resistance to systemic bacterial infections^4–11^. Delays or disruptions to maternal microbial transmission, as is common among cesarean delivered newborns and antibiotic-exposed neonates, is associated with increased risk of sepsis, immune system hypersensitivity, allergy, asthma, altered growth trajectories, and obesity^12–14^.

The vaginal microbiota represents a primary maternal microbial reservoir for the colonization of the newborn during a vaginal birth^3,15–19^. In contrast to the gut microbiome, the vaginal microbiota in reproductive age individuals comprises of minimally diverse communities that are divided into five primary community state types (CST)^20–25^. CST I, II, III, and V are dominated by the presence of *Lactobacillus* species and represent the most common communities in the cervicovaginal space. An additional vaginal community state type, CST IV, is defined by an absence of *Lactobacillus* and the presence of anaerobic bacteria, including *Snethia, Prevotella, Megasphaera, Gardnerella vaginalis*, and *Atopobium vaginae*^20–22,26–29^. Strain-resolved analyses showed that maternal vaginal bacteria, including *Lactobacillus crispatus*, *G. vaginalis*, and *A. vaginae*, are among the earliest colonizers to persist within the infant intestinal tract during the first few days after birth^3^. While these members of CST I (*L. crispatus*) and CST IV (*G. vaginalis, A. vaginae*) exhibit distinct metabolic and immune properties in the female reproductive tract^12,27,28^, the possible functional consequences of transferring these communities from mother to the newborn is not clear^3^. Further, given the increasing scientific interest and debate regarding the safety and efficacy of seeding cesarean delivered newborns with maternal vaginal or fecal microbiota, establishing preclinical models of birth-associated microbial exposure may provide additional mechanistic insight on whether these practices are associated with positive or negative neonatal outcomes^17,18,30–35^.

Thus, to determine how birth-associated exposure to maternal vaginal microbiota exhibit community-specific effects on offspring outcomes, we first established a protocol that simulates birth-associated exposure in humans by oral gavaging cesarean delivered mice with vaginal microbiota samples collected from women late in pregnancy. While there are numerous microbial communities transferred from mother to newborn that exhibit complex combinatorial effects, we employed a reductionist experimental strategy by focusing on vaginal microbial communities to which a newborn would be exposed during vaginal birth and persist in the newborn within the first days of life. Comparisons focused on the *Lactobacillus crispatus*-dominant community state type (CST I) and the community state type that lacks *Lactobacillus* dominance and is replaced by anaerobic bacteria, including *G. vaginalis* and *A. vaginae* (CST IV)^22,24,36,37^. Following technical validation and optimization, we determined whether there were CST-specific effects on key aspects of postnatal gut and immune development, growth and metabolism, circulating immunity, and neural circuits involved in the control of feeding and metabolism in C-section delivered and inoculated mice. Additionally, development of mucosal tissues and the immune system occurs during the prenatal period, and the developmental programming of these tissues is exquisitely sensitive to alterations in the maternal milieu during pregnancy^38–43^. Driven by these insights, we hypothesized that perturbations to the prenatal environment would influence how offspring respond to postnatal microbial exposure. To examine this hypothesis, we used a separate mouse model to determine whether single or compounding prenatal risk factors—diet-induced obesity and the presence of *G. vaginalis*—impact prenatal endpoints and the postnatal response to the colonizing microbiota.

## Results

### Classification and selection of human vaginal microbiota from women across pregnancy

Human vaginal secretions were self-collected using the Copan ESwab^TM^ collection and transport system during pregnancy by women prospectively enrolled in the Birth, Eating, and the Microbiome study conducted at the University of Maryland, Baltimore. Samples were collected weekly until delivery. Community state types were established using VALENCIA (*VA*gina*L* community state typ*E N*earest *C*entro*I*d cl*A*ssifier), a nearest centroid classification method for vaginal microbial communities based on composition^20^. Of note, the VALENCIA classification method was used to determine CST membership that was then used to select samples for the inoculation studies^20^. VALENCIA itself was not used for any of the analyses in this paper. Additional sample selection criteria for mouse transplantation experiments included: (1) sequencing-based and cultivation-dependent confirmation of CST I or CST IV vaginal microbiota; (2) availability of samples between 36 and 39 weeks of pregnancy to capture the microbial communities with the greatest relevance toward what would be vertically transferred to newborns during birth; and (3) women remained in their respective CST throughout pregnancy.

### Establishment of a human microbiota-associated mouse model of birth-associated exposure to human vaginal microbiota

**Fig. 1:**
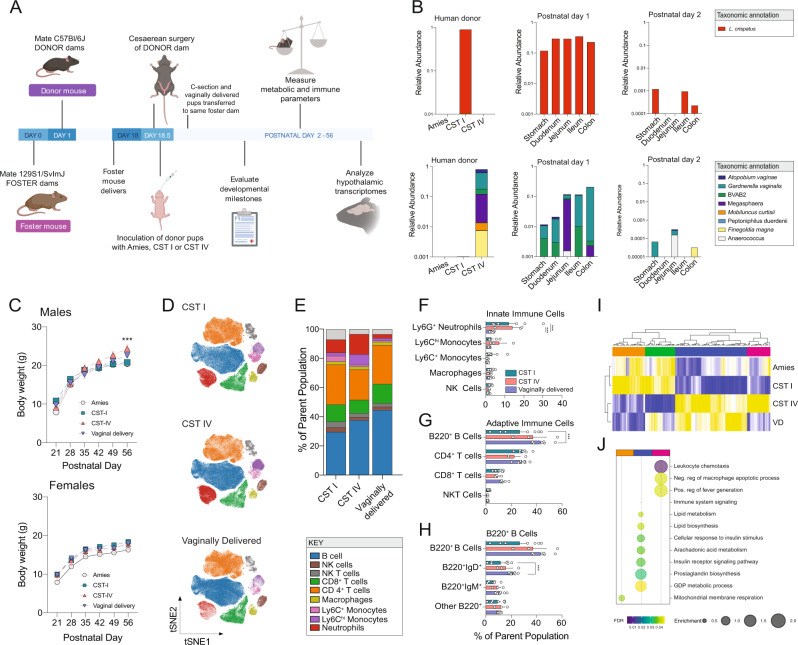
Exposure at birth to distinct maternal microbiota on offspring growth, circulating immunity, and hypothalamic gene expression patterns. **A** Schematic of the experimental timeline and protocol used to establish a mouse model to investigate the impact of exposure to maternal human vaginal microbiota at birth on lasting offspring outcomes. See Methods and accompanying Supplementary Fig. 1 for additional details. Created with BioRender.com. **B** Validation of presence of human vaginal microbiota across intestinal segments of C-section neonate mice inoculated with *L. crispatus-*dominated microbiota (CST I) or lactobacilli-deficient, nonoptimal microbiota (CST IV) collected from late-gestation women using 16 S rRNA gene marker sequencing. Upper left panel, mean relative abundance of *L. crispatus* showing no detectable amplification of *L. crispatus* in the Amies transport medium and CST IV samples. Upper middle and right panel, mean relative abundance of *L. crispatus* in CST I inoculated offspring at postnatal day 1 and 2. No amplification of *L. crispatus* was detected in Amies and CST IV inoculated pups at either timepoint. Lower left panel, mean relative abundance from CST IV associated taxa showing no detectable amplification in the Amies transport medium and CST I samples. Upper middle and right panel, mean relative abundance of CST IV inoculated offspring at postnatal day 1 and 2. No amplification of CST IV was detected in Amies, and CST I inoculated pups at either timepoint. *N* = 7–20 per group and intestinal segment. **C** Sex-specific effects of CST I and CST IV inoculation on body weight across development. Top panel, male body weight was significantly changed over time (two-way ANOVA, main effect of time F_5, 170_ = 689.4, *P* < 0.0001) and the interaction between time and treatment (two-way ANOVA, time*treatment interaction, F_15, 170_ = 8.544, *P* < 0.0001). Tukey’s post-hoc analysis revealed that CST IV males weighed more than Amies and CST I males at P56 (Tukey’s, *P* = 0.0009 and 0.0006, respectively). No differences in body weight in CST I males compared with Amies or vaginally delivered males at P56 (Tukey’s, *P* = 0.990 and *P* = 0.261). Bottom panel, female body weight was significantly changed over time (two-way ANOVA, main effect of time F_5, 130_ = 306.819, *P* < 0.0001). No effect of treatment or their interaction was observed. Males: *N* = 7 Amies, 12 CST I, 11 CST IV, 7 vaginally delivered per timepoint. Females: *N* = 8 Amies, 9 CST I, 7 CST IV, 6 vaginally delivered per timepoint. Data represented as mean ± SEM. Data are representative of two independent experiments. ***P < 0.001. **D**–**F** High-dimensional, single-cell mapping reveals lasting effects of CST I and CST IV inoculation on the circulating immune compartment in adult males. **D** *t-*Distributed stochastic neighbor embedding (t-SNE) visualization demonstrating CyTOF assessment of CD45+ immune cells in whole blood of CST I, CST IV, and vaginally delivered postnatal day 56 adult males (equal sampling across treatment groups, total 390,000 events). **E** Average frequencies of major leukocytes within whole blood in CST I, CST IV, and VD adult males. **F** Frequencies of circulating innate immune cells showing increased frequency of neutrophils in CST I and CST IV males compared with VD males at P56 (two-way ANOVA, treatment*immune cell interaction, F_8, 56_ = 2.870, *P* = 0.0095; Tukey’s post-hoc CST I vs VD *P* = 0.0001; Tukey’s post-hoc CST IV vs VD *P* = 0.0003). *N* = 7 CST I males, 3 CST IV males, 7 vaginally delivered males. Data represented as mean ± SEM with individual data points overlaid. ***P < 0.001. **G** Frequencies of circulating adaptive immune cells showing decreased frequency of B220 + B cells in CST I males compared with VD males at P56 (two-way ANOVA, treatment*immune cell interaction, F_6, 56_ = 2.492, *P* = 0.0330; Tukey’s post-hoc CST I vs. VD *P* = 0.0003). *N* = 7 CST I males, 3 CST IV males, 7 vaginally delivered males. Data represented as mean ± SEM with individual data points overlaid. ***P < 0.001. **H** Frequencies of circulating B220^+^ B cells showing decreased frequency of B220^+^IgD^+^ B cells in CST I males compared with VD males (two-way ANOVA, treatment*immune cell interaction, F_4, 28_ = 5.471, *P* = 0.0022; Tukey’s post-hoc CST I vs. VD *P* = 0.0017). *N* = 7 CST I males, 3 CST IV males, 7 vaginally delivered males Data represented as mean ± SEM with individual data points overlaid. ***P < 0.001. **I** Heatmap depicting mean expression of genes in the paraventricular nucleus of the hypothalamus in P56 males. Paralleling body weight differences between CST I and CST IV males at P56, RNA-seq analysis shows differences in PVN gene expression patterns between CST I and CST IV males (linear fit model, FDR < 0.1, log(FC) = 1.5). Unbiased hierarchical clustering showing similarity in transcriptional patterns between CST IV and VD males, and CST I and Amies males. Color based on row *Z*-scores for each gene. Three clusters of differentially regulated genes in each treatment group are indicated. *N* = 4–6 males per treatment. **J** Cluster-based functional enrichment analysis of differentially expressed genes in the paraventricular nucleus of the hypothalamus in P56 males. Significant enrichment of pathways involved in metabolism and immunity in CST IV compared with CST I males, showing unique pathways in the PVN that are associated with body weight changes in CST IV males (FDR < 0.05). Bubble plot size denotes enrichment. Clusters identified in differential gene expression analysis were used for this pathway enrichment and color-coded as blue (Cluster 1), orange (Cluster 2) and pink (Cluster 3). *N* = 4–6 males per treatment.

We previously established a C-section and oral gavage protocol to manipulate the composition of the colonizing microbiota at birth in mice^44^. We sought to apply this method within a translational context to investigate the role of distinct human vaginal community state types on offspring outcomes. Specific pathogen free C57BL/6 J mouse pups were cesarean delivered on embryonic (E) day 18.5 and inoculated with CST I or CST IV samples by oral gavage^44^ (Supplementary Fig. 1, Fig. 1A). Several controls were included in these experiments. As a control for the oral gavage, a cohort of C-section delivered pups were inoculated with Amies, the transport medium in which the human vaginal swab samples were stored. Age-matched vaginally delivered (VD) offspring were also included in experiments to control for mode of delivery and premature birth (i.e., C-section offspring are born 0.5 days early). One male and one female pup representative from every treatment group was cross-fostered to treatment naïve 129/SvJ surrogate dams as a control for cage effects and the postnatal environment on offspring phenotype^45,46^. The 129/SvJ strain was chosen based on low occurrence of infanticide and acceptance of nongenetically related offspring when compared with the C57BL/6 J strain^47^. Following inoculation, tattooing, and transfer to foster dam, cross-fostered offspring remained undisturbed with the foster dam and littermates. This approach permits a modular method to manipulate the prenatal environment, the postnatal environment and maternal microbial communities to assess their singular or combinatorial contribution to offspring health outcomes.

To confirm detection of human vaginal microbiota in intestinal segments of C-section delivered and inoculated mouse pups, we used microbiome profiling by targeted sequencing of the V4 region of the 16 S rRNA gene and quantitative PCR as two independent methods of technical validation. Analysis of microbiome profiling data from intestinal segments collected 24 h post-inoculation revealed no amplification of CST IV taxa in pups inoculated with CST I, and CST IV inoculated offspring showed no amplification of CST I taxa (Fig. 1B). No amplification of CST I or CST IV taxa was observed in Amies inoculated offspring. Consistent with 16 S rRNA data, we confirmed presence of *G. vaginalis* via qPCR in CST IV inoculated pups but not in CST I, Amies, or vaginally delivered pups (Supplementary Fig. 2a).

Detection of human vaginal microbiota declined by 48 h following C-section and inoculation (Fig. 1B). To identify microbial sources that may contribute to this subsequent wave of colonization, environmental and maternal samples were collected across various body sites and assessed using microbiome profiling (Supplementary Fig. 3a). Nonmetric multidimensional scaling (NMDS) analysis of maternal and environmental samples showed that environmental and maternal samples formed unique and nonoverlapping clusters (Supplementary Fig. 3b). Comparison of taxa between maternal and environmental samples revealed site-specific enrichment of microbiota, including *Pasteurella* recovered from maternal skin and vaginal fluid, while *Actinobacillus muris* was recovered from maternal skin (Supplementary Fig. 3c). Samples from the intestinal tract of C-section offspring and environmental samples formed nonoverlapping clusters, suggesting that the local environment is not at this sampled timepoint a significant reservoir of microbiota that colonize C-section delivered offspring (Supplementary Fig. 3d). Microbial composition recovered from maternal skin showed similarity with taxa recovered from offspring intestinal segments, with *Pasteurella* identified as the most abundant taxa present in all offspring at postnatal day 2 (Supplementary Fig. 3e–h). Differential abundance analysis showed increased abundance of *Gammaproteobacteria* and *Escherichia-Shigella* in the intestinal tract of CST IV pups compared with CST I pups 48 h following C-section and inoculation (Supplementary Fig. 3i, j).

Lastly, to determine whether birth-associated exposure to defined communities of human vaginal microbiota have a lasting impact on offspring microbial composition in adulthood, a fecal sample was collected from Amies, CST I and CST IV inoculated male littermates in adulthood and analyzed using 16 S rRNA marker gene sequencing. Measures of community structure and diversity were similar between all treatment groups by adulthood, consistent with well established dominant effects of the local cage environment on the fecal microbiota of littermates (Supplementary Fig. 4)^46,48^.

### Human microbial exposure contributes to lasting sex-specific changes in growth, circulating immunity, and hypothalamic transcriptional profiles

We next determined how birth-associated exposure to CST I or CST IV might influence key phenotypes shown to be modulated by the microbiota^49^, including body weight, immunity and the brain^50–52^. Body weight measurements from weaning to adulthood showed that the growth curve for CST IV-exposed males was steeper than that of CST I and vehicle males, but not different from vaginally delivered males (Supplementary Fig. 1, Fig. 1C). CST IV males showed differences in percentage of body weight gain and adult body weights compared with CST I and Amies males (Fig. 1C and Supplementary Fig. 5a, b). Body weight differences were independent of body length, as no differences in rump-snout length were observed between the four groups (Supplementary Fig. 5c). No differences in body weight or body length were observed between the four groups in female offspring (Fig. 1C and Supplementary Fig. 5d-f). Based on the sex-specific effects of human vaginal microbiota exposure on body weight and size, the proceeding experiments focused on male offspring.

We next determined whether birth-associated microbial exposure had a lasting impact on circulating immune cell populations in adult males using single-cell mass cytometry time of flight (CyTOF)^53^. Differential abundance analysis revealed that CST I and CST IV males showed a decrease in the frequency of neutrophils compared with vaginally delivered males, while the frequency of total B cells and IgD^+^ B cells were decreased in CST I males (Fig. 1D-H and Supplementary Fig. 6a-c). This may suggest that birth-associated colonization by distinct human microbial communities is associated with shifts in peripheral immune cell composition in a CST-specific manner.

To determine whether the peripheral outcome measures body weight and circulating immunity were associated with changes to the central nervous system, we compared gene expression patterns of the paraventricular nucleus of the hypothalamus (PVN) between adult Amies, CST I, CST IV, and VD males using bulk RNA sequencing. The PVN was chosen based on the well established role of this hypothalamic nuclei in the maintenance of growth, body weight and immunity^54–58^. Differential gene expression analysis (FDR < 0.1, Log(FC) > 1.5) comparing CST I and CST IV males identified 175 genes with altered expression in the PVN (Fig. 1L). Unbiased hierarchical clustering of the differentially expressed genes revealed clustering between Amies and CST I males and clustering between CST IV and VD males, paralleling the growth curve and body weight differences observed in these males (Fig. 1C, L). Further, cluster-based functional enrichment revealed upregulation of pathways involved in lipid metabolism, insulin receptor signaling, and immune activation in the PVN in CST IV males compared with Amies, CST I, and VD males (Fig. 1J). Collectively, these results suggest that birth-associated exposure to human vaginal microbiota is associated with metabolic, immune, and neural outcomes in adult mice in a CST- and sex-specific manner.

### Exposure to human vaginal microbiota influences transcriptional patterns in the mouse postnatal ileum

**Fig. 2:**
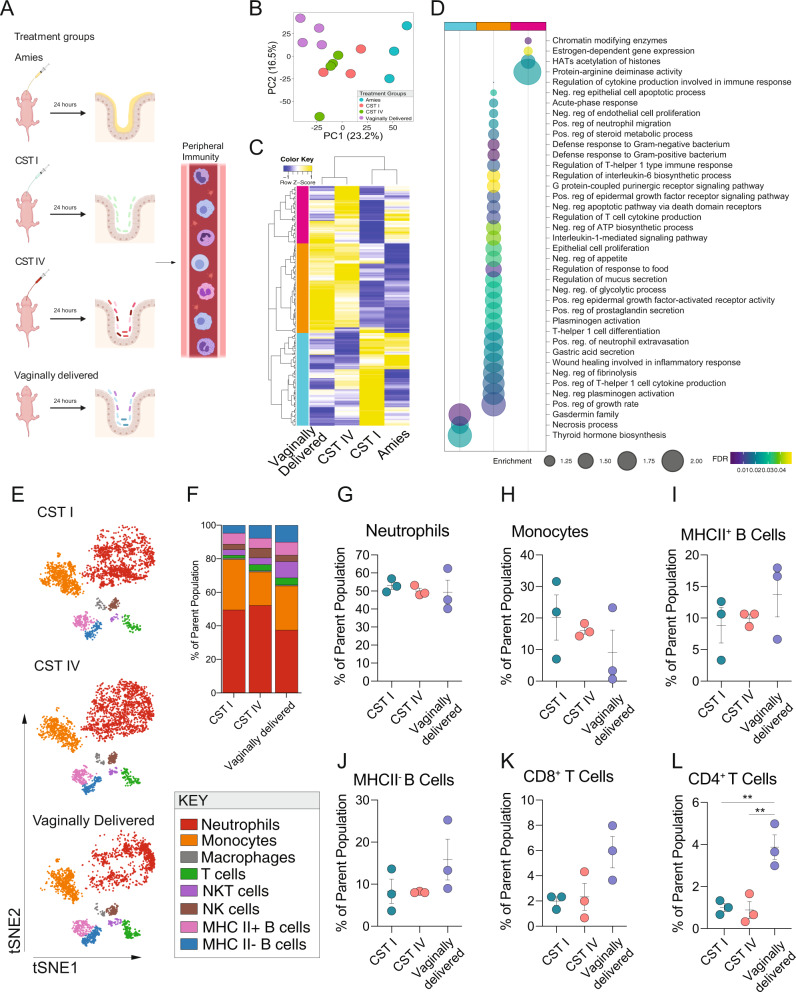
Transcriptional landscape of the neonatal ileum and the circulating immune compartment is influenced by vaginal community state type. **A** Schematic of experimental outline to determine the influence of the human microbiome on transcriptional profiles of the neonatal ileum and the circulating immune compartment. Created with BioRender.com. **B** Principal component analysis plots of gene expression data demonstrating the distribution of P1 male ileum samples colored by treatment group, revealing clustering is driven by developmental maturity, mode of delivery, and colonization (C-section Amies inoculated vs. vaginally delivered) and the C-section pups inoculated with human vaginal microbiota as an intermediate between the Amies and vaginally delivered pups. P1 corresponds to 24 hrs post-inoculation. *N* = 3–4 males per treatment. **C** Heatmap depicting mean expression of genes in the ileum P1 males, showing differences between CST I and CST IV males (linear fit model, FDR < 0.1, log(FC) = 1.5). Unbiased hierarchical clustering showing similarity in transcriptional patterns between CST IV and VD males, and CST I and Amies males. P1 corresponds to 24 h post-inoculation. Color based on row *Z*-scores for each gene. Three clusters of differentially regulated genes in each treatment group are indicated. *N* = 3–4 males per treatment. **D** Cluster-based functional enrichment analysis of differentially expressed genes in the ileum of P1 males showing significant enrichment of pathways involved in defense response to bacteria, innate immune activation, chemotaxis, and mucus secretion in the ileum of CST IV relative to CST I inoculated males (FDR < 0.05). Bubble plot size denotes enrichment. *N* = 4–6 males per treatment. **E** t-SNE visualization demonstrating CyTOF phenotyping of CD45+ immune cells in whole blood of CST I, CST IV, and vaginally delivered males showing neutrophils as the major immune subset in the circulating immune compartment at P1 (equal sampling across treatment groups, total 10,000 events). P1 corresponds to 24 h post-inoculation. *N* = 3 males per treatment. **F** Average frequencies of major leukocytes within whole blood in P1 CST I, CST IV, and VD males. *N* = 3 males per treatment. **G**–**L** Frequencies of circulating (**G**) neutrophils, (**H**) monocytes, (**I**) MHC II^+^ B cells, (**J**) MHC II^-^ B cells, **K** CD8 + T cells, and **L** CD4^+^ T cells within whole blood in P1 CST I, CST IV, and VD males. (1) Frequency of CD4^+^ T cells were significantly increased in VD males compared with CST I and CST IV males (One-way ANOVA, F_2,6_ = 15.97, *P* = 0.0040; Tukey’s post-hoc CST I vs. VD *P* = 0.0072; CST IV vs. VD *P* = 0.0059). *N* = 3 males per treatment. Data represented as mean ± SEM with individual data points overlaid. **P < 0.01.

Our observation that birth-associated colonization by distinct vaginal community state types influenced adult outcomes prompted us to determine whether exposure to these communities may produce early-life programmatic changes that may associate with these lasting outcomes. The female reproductive tract colonized by a high-diversity *Lactobacillus*-deficient bacterial community, such as CST IV, is associated with alterations to transcriptional networks, higher local proinflammatory cytokine levels, and activation of antigen-presenting cells via lipopolysaccharide sensing pathways when compared with presence of CST I in the female reproductive tract^59–61^. We hypothesized that birth-associated exposure to human vaginal microbiota may produce similar CST-specific effects in the neonatal intestinal tract^62^. Twenty-four hours following C-section and inoculation, the ileum was collected from Amies, CST I, CST IV, and age-matched VD males, and gene expression patterns were analyzed by bulk RNA sequencing (Fig. 2A). A principal components analysis showed that Amies and VD males formed distinct clusters, while CST I and CST IV males represented an intermediate phenotype between Amies and VD male offspring (Fig. 2B). Differential gene expression analysis (FDR < 0.1, Log(FC) > 1.5) comparing CST I and CST IV males identified 128 genes with altered expression in the ileum (Fig. 2C). Unbiased hierarchical clustering of the differentially expressed genes revealed clustering between Amies and CST I males and clustering between CST IV and VD males, showing that exposure to CST I or CST IV is associated with unique gene expression patterns in the ileum (Fig. 2C). Cluster-based functional enrichment revealed significant enhancement of metabolic and immune pathways in the ileum of Amies, CST I, CST IV, and VD males (Fig. 2D). The CST I-enriched cluster is enriched in gene pathways involved in the activation of gasdermin family, necrosis regulation, and stimulation of hormone synthesis. Conversely, the two clusters shared by CST IV and VD males included enrichment of gene pathways involved in the regulation of mucus secretion, defense response to bacteria, cholesterol metabolism, neutrophil migration, and chromatin modification (Fig. 2D). These results suggest that exposure to human vaginal microbiota is associated with CST-specific transcriptional patterns in the neonate ileum at this specific period of development.

### Exposure to human vaginal microbiota influences neonatal circulating immune cell composition

A surge in circulating neutrophils occurs in human and murine newborns within 72 h of postnatal life, and overlaps with birth-associated exposure to maternal microbiota^3,63^. To confirm that this surge in neutrophils is dependent on exposure to maternal microbiota, we used an established model of perinatal antibiotic exposure whereby pregnant dams are exposed to ampicillin, vancomycin, and neomycin (all 1 mg/ml) from gestational day 15.5–18.5 to deplete microbiota and prevent vertical transmission of microbiota^64^. On embryonic day 18.5, offspring from antibiotic-exposed donor dams were cesarean delivered and male offspring were colonized with CST I or CST IV. Age-matched VD male offspring were also included to account for potential effects of premature birth, mode of delivery, and antibiotic exposure. Hematology analysis revealed that circulating neutrophils were significantly reduced in antibiotic-exposed male offspring compared with CST I, CST IV, and VD male offspring, replicating previous work showing that exposure to maternal microbiota is associated with an increase in circulating neutrophils shortly after birth^64^. Further, no differences were observed in circulating neutrophil counts between CST I, CST IV, and VD male offspring (Supplementary Fig. 7a). Hematology analysis also revealed an increase in basophil counts in antibiotic-exposed male offspring compared with CST IV and VD male offspring, but not CST I offspring (Supplementary Fig. 7b).

A limitation of the hematology analysis is the inability to further classify granulocyte and lymphocyte populations into specific immune subsets. Thus, to attain a high-resolution snapshot of circulating immune cell compartment in neonates, whole blood from 1-day-old males was analyzed by CyTOF (Fig. 2E-L, and Supplementary Fig. 8a-c). Consistent with the hematology analysis, neutrophils represented the most abundant immune cell subset in circulation in 1-day-old males (Fig. 2G). Phenotypic analysis of neutrophils revealed a CD14+ neutrophil subcluster present in all samples (Supplementary Fig. 8a-c). Albeit at lower frequencies, additional innate and adaptive immune cell populations were detected in circulation of 1-day-old CST I, CST IV, and VD male offspring, including monocytes, B cells, macrophages, natural killer cells, CD8^+^ T cells, and CD4^+^ T cells (Fig. 2H-L). Comparison of immune cell frequencies revealed increased frequency of CD4^+^ T cells in the circulation of VD males compared with CST I and CST IV males, an observation that may reflect developmental differences due to mode of delivery or premature birth in the cesarean delivered and inoculated male offspring compared with vaginally delivered offspring (Fig. 2L). Together, our analysis of the circulating immune compartment revealed immune cell populations that expand and contract in response to birth-associated exposure to human microbial communities, and immune cell populations that may be influenced by premature birth and mode of delivery in mice.

### Maternal environmental factors influence postnatal offspring response to the colonizing microbiota

It is well established that maternal exposures during pregnancy, such as stress, infection, or malnutrition^7^, impact prenatal development, and increase offspring vulnerability to subsequent environmental stressors across the lifespan^39,65–67^. The possibility that prenatal exposures may influence postnatal response to maternal microbiota is not known in this model. To test this hypothesis, we established a two-hit model in which offspring were exposed prenatally to a nonoptimal maternal diet or vaginal microbiota, or both, and then were assessed following colonization.

**Fig. 3:**
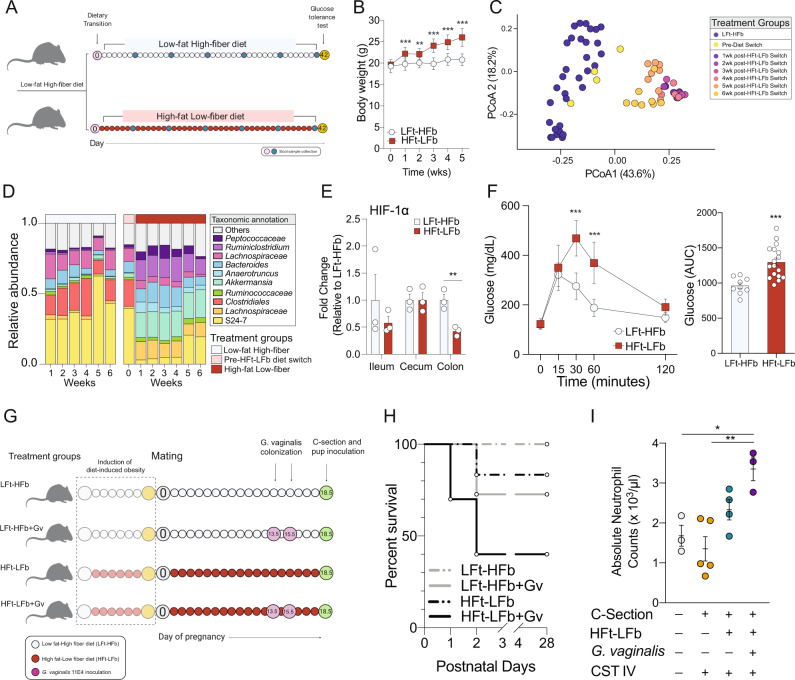
Modeling compounding maternal risk factors results in an adverse response to birth-associated microbial exposure. **A** Schematic of experimental timeline for the induction of pregestational excessive weight gain, glucose intolerance, and microbiota alterations through the consumption of a high-fat low-fiber diet (HFt-LFb). Day 0 refers to the beginning of the dietary treatment and does not reflect chronological age. Created with BioRender.com. **B** Consumption of a high-fat low-fiber diet accelerates body weight gain in females compared with females consuming a low-fat high-fiber diet (two-way ANOVA, main effect of time, F_5, 120_ = 94.17, *P* < 0.0001; main effect of diet, F_1, 24_ = 30.70, *P* < 0.0001; time*diet interaction, F_5, 120_ = 36.61, *P* < 0.0001). *N* = 12 LFt-HFb females, 20 HFt-LFb females per timepoint. Data represented as mean ± SEM. Time (in weeks) is measured from time of diet switch. Data are representative of two independent experiments. **P < 0.01, ***P < 0.001. **C** Principal coordinates analysis demonstrating temporal dynamics of diet on the gut microbiota, whereby 1wk consumption of a high-fat low-fiber diet resulted in separate clustering from females consuming a low-fat high-fiber diet that failed to recover during the treatment window. *N* = 12 LFt-HFb females, 20 HFt-LFb females per timepoint, total of 68 samples that passed quality filtering. **D** Mean relative abundance of top ten taxa showing rapid changes to the fecal microbiota following transition to a high-fat low-fat diet, characterized by a loss of Clostridiales. *N* = 12 LFt-HFb females, 20 HFt-LFb females per timepoint, total of 68 samples that passed quality filtering. **E** Expression of HIF-1a is significantly decreased in the colon following 6-week consumption of a high-fat low-fiber diet relative to females consuming a low-fat high-fiber diet (two-sided *t*-test, *t*_*4*_ = 4.9, *P* = 0.008), indicating possible disruption in hypoxia homeostasis in the colon. *N* = 3 LFt-HFb females, 3 HFt-LFb females. Data represented as mean ± SEM with individual data points overlaid. **P < 0.01. **F** Left, Plasma glucose levels during a glucose tolerance test in females consuming either a high-fat low-fiber or low-fat high-fiber diet. Females consuming a high-fat low-fiber diet showed significant delay in glucose clearance (two-way ANOVA, main effect of time, F_4, 108_ = 106.33, *P* < 0.0001; main effect of diet, F_1, 27_ = 15.86, *P* = 0.0005; time*diet interaction, F_4, 108_ = 15.44, *P* < 0.0001). Right, AUC of total plasma glucose levels showing increase glucose levels in females consuming a high-fat low-fiber diet (two-tailed *t*-test, *t*_24_ = 4.055, *P* = 0.005). *N* = 9 LFt-HFb females, 17 HFt-LFb females Data represented as mean ± SD with individual data points overlaid. ***P < 0.001. **G** Schematic of experimental design to determine the impact of prenatal exposure to compounding maternal adversities, such as diet and presence of a common member of CST IV, during pregnancy on offspring outcomes. We induced the pregestational phenotype and colonized pregnant females to *G. vaginalis* 11E4 (Gv) gestational day 13.5 and 15.5. At gestational day 18.5, offspring from all treatment groups were colonized with the nonoptimal CST IV human vaginal microbiota. Created with BioRender.com. **H** Survival of offspring from dams that experience a single or multiple compounding adversities. All pups were C-section delivered and gavaged with human CST IV inoculant, showing the highest offspring mortality risk in HFt-LFb+Gv offspring that were exposed to CST IV at birth. LFt-HFb = low-fat high-fiber; HFt-LFb = high-fat low-fiber; Gv = *G. vaginalis* 11E4. Kaplan–Meier survival analysis. *N* = 20 offspring per treatment condition. **I** Compounding effects of maternal diet, maternal vaginal colonization by *G. vaginalis* (Gv), and exposure to CST IV on absolute number of neutrophils in the circulation of postnatal day 1 male pups. Triple-hit male pups show significant increase in neutrophils compared with vaginally delivered and CST IV inoculated offspring (One-way ANOVA, main effect of treatment, F_3, 11_ = 8.452, *P* = 0.0034; VD vs. Triple Hit, *P* = 0.0188; CST IV vs. Triple Hit, *P* = 0.0026). *N* = 3 vaginally delivered males, 5 CST IV inoculated LFt-HFb males, 4 CST IV inoculated HFt-LFb males, 3 CST IV inoculated HFt-LFb+Gv males. Data represented as mean ± SEM with individual data points overlaid. *P < 0.05, **P < 0.01.

To validate the two-hit model, we first confirmed the effects of consuming a high-fat low-fiber diet on key maternal outcomes before and during pregnancy. An independent cohort of naïve female mice were provided a high-fat low-fiber (HFt-LFb) diet or continued to consume the low-fat high-fiber (LFt-HFb) diet (Fig. 3A). Weekly body weight measurements over a 6-week period showed a significant body weight increase in HFt-LFb females compared with LFt-HFb females (Fig. 3B). Body weight changes accompanied significant shifts in gut microbiota structure and composition, characterized by an enduring loss of the immunomodulatory and soluble fiber-fermenting members of the class *Clostridia* and a bloom of the mucin-degrading *Akkermansia* in HFt-LFb relative to LFt-HFb females (Fig. 3C, D). Differential abundance analysis revealed disruption in the abundance of 48 taxa between HFt-LFb and LFt-HFb females (FDR > 0.05). Consistent with previous work showing dysregulation of intestinal hypoxia due to high-fat low-fiber diet consumption, we found decreased expression of the hypoxia-inducible factor-1-alpha in the colon of HFt-LFb relative to LFt-HFb females (Fig. 3E)^68–70^. Glucose clearance was also significantly delayed in HFt-LFb relative to LFt-HFb females following administration of a glucose tolerance test, replicating well established effects of diet-induced obesity on glucose tolerance (Fig. 3F)^71,72^.

During pregnancy, excessive body weight gain persisted in HFt-LFb dams from gestational day 0.5–14.5 relative to LFt-HFb dams. Differences in body weight disappeared between gestational day 15.5–18.5, and was attributed to a change in the trajectory of daily body weight gain in HFt-LFb dams relative to LFt-HFb dams (Supplementary Fig. 9a). In parallel to the body weight gain in HFt-LFb dams, daily tracking of the maternal gut microbiota revealed significant alterations to microbiota structure and composition in HFt-LFb dams compared with LFt-HFb dams across pregnancy (Supplementary Fig. 9b, c). Differential abundance analysis revealed 36 differentially abundant taxa between HFt-LFb and LFt-HFb, including the reduced relative abundance of butyrate-producing *Butyricoccus* and the immunomodulatory *Candidatus Arthromitus* in HFt-LFb compared with LFt-HFb pregnant dams (Supplementary Fig. 9d, e)^73,74^.

Following confirmation of the impact of the single-hit dietary exposure on maternal body weight, glucose tolerance, and the gut microbiota, we next examined the effect of a nonoptimal vaginal microbiota on these outcomes. Pregnant HFt-LFb or LFt-HFb dams were intravaginally inoculated with 2 × 10^8^ colony forming units of *G. vaginalis* strain 11E4^75–79^. This bacterial species was chosen based on previous work showing that *G. vaginalis* (Gv) is a common member of CST IV, induces immune activation and inflammation in the vaginal epithelium, and contributes to the pathogenesis of bacterial vaginosis in humans and rodent models^77,79,80^. Inoculating HFt-LFb or LFt-HFb dams with *G. vaginalis* 11E4 at gestational day 13.5 and 15.5 did not impact body weight or gut microbiota (Fig. 3A-F and Supplementary Fig. 9a). As further validation, we confirmed presence of *G. vaginalis* in the vaginal fluid of inoculated compared with noninoculated dams by qPCR (Supplementary Fig. 10a).

We next determined whether prenatal exposure to maternal diet-induced obesity and a nonoptimal vaginal microbiome, alone or in combination, affected the offspring postnatal response to birth-associated exposure to human vaginal microbiota. On embryonic day 18.5, offspring from dams consuming LFt-HFb, LFt-HFb dams colonized with Gv (LFt-HFb+Gv), HFt-LFb, and HFt-LFb+Gv were C-section delivered, exposed to CST IV, transferred to foster dams, and monitored for litter acceptance and survival (Fig. 3G). Following colonization with CST IV, HFt-LFb+Gv male offspring showed a 60% mortality rate compared with the 29% mortality in LFt-HFb+Gv males, 17% in HFt-LFb males, and no mortality in LFt-HFb at postnatal day (P) 3 (Fig. 3H). No additional mortality was observed beyond P3. To determine whether increased offspring mortality was specific to CST IV, we also transplanted CST I into a separate cohort of offspring exposed to the same combinations of maternal diet and *G. vaginalis* vaginal colonization. We observed that HFt-LFb+Gv male offspring colonized with CST IV exhibited a mortality rate of 60% while HFt-LFb+Gv male offspring colonized with CST I exhibited a mortality rate of 50%, demonstrating a 10% difference in the survival rate among high-risk offspring exposed to CST I in this mouse model (Supplementary Fig. 11a).

We next examined the possibility that morbidity phenotype in HFt-LFb+Gv male offspring exposed to maternal microbiota was related to neutrophil counts in circulation. Twenty-four hours following C-section delivery and inoculation, HFt-LFb+Gv male offspring exposed to CST IV showed a significant increase in circulating neutrophil counts compared with LFt-HFb males exposed to CST IV and VD males, but not HFt-LFb males exposed to CST IV (Fig. 3I). Absolute neutrophil counts more than doubled in HFt-LFb+Gv males exposed to CST IV (Mean = 3.5 × 10^3^ per µL of blood) when compared with LFt-HFb males exposed to CST IV (Mean = 1.5 × 10^3^ per µL of blood), consistent with a clinical definition of neonatal neutrophilia^81,82^.

### Altered gene expression patterns in the placenta and fetal ileum is associated with adverse neonatal outcomes following microbial colonization

**Fig. 4:**
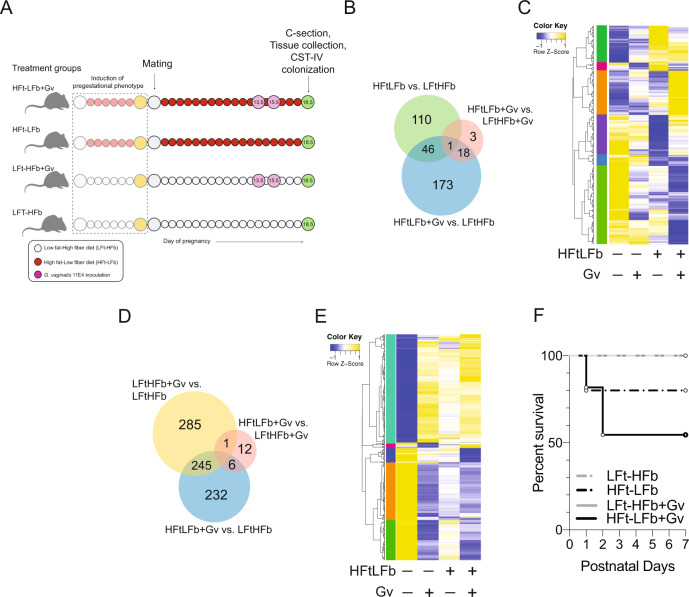
Prenatal exposure to maternal diet-induced obesity and a nonoptimal vaginal microbiota alter the transcriptional landscape in the placenta and fetal ileum of male offspring. **A** Schematic of experimental design to determine whether compounding maternal adversities, such as diet and *G. vaginalis* vaginal colonization, impact fetal development that may contribute to increased offspring mortality risk. We induced the pregestational phenotype and dams were colonized with *G. vaginalis* 11E4 on gestational day 13.5 and 15.5. At gestational day 18.5, tissue from one cohort of male offspring was collected for analysis of gene expression patterns in the placenta and ileum. A second cohort of offspring were colonized with the nonoptimal CST IV human vaginal microbiota. Created with BioRender.com. **B** Venn diagram displaying the number of differentially expressed genes in the embryonic day 18.5 placenta of male offspring exposed to a maternal high-fat low-fiber diet, *G. vaginalis* 11E4 vaginal colonization, or a combination (linear fit model, FDR < 0.1, log(FC) = 1.5; *n* = 351 genes). LFt-HFb = low-fat high-fiber; HFt-LFb = high-fat low-fiber; Gv = *G. vaginalis* 11E4. **C** Heatmap depicting mean expression of genes in the placenta of embryonic day 18.5 male offspring exposed to a maternal high-fat low-fiber diet, *G. vaginalis* 11E4 vaginal colonization, or a combination (HFt-LFb+Gv) (linear fit model, FDR < 0.1, log(FC) = 1.5). These comparisons reveal a unique cluster of genes that are differentially expressed in HFt-LFb+Gv male placenta compared with other treatment groups. *Z*-scores plotted across individuals for each gene. LFt-HFb = low-fat high-fiber; HFt-LFb = high-fat low-fiber; Gv = *G. vaginalis* 11E4. Color based on row *Z*-scores for each gene. Six clusters of differentially regulated genes in each treatment group are indicated. *N* = 3 males per treatment. **D** Venn diagram displaying the number of differentially expressed genes in the embryonic day 18.5 ileum of male offspring exposed to a maternal high-fat low-fiber diet, *G. vaginalis* 11E4 vaginal colonization, or a combination (HFt-LFb+Gv) These comparisons reveal a unique cluster of genes that are differentially expressed in the fetal ileum of HFt-LFb+Gv males compared with other treatment groups (linear fit model, FDR < 0.1, log(FC) = 1.5; *n* = 781 genes). LFt-HFb = low-fat high-fiber; HFt-LFb = high-fat low-fiber; Gv = *G. vaginalis* 11E4. **E** Heatmap depicting mean expression of genes in the ileum of embryonic day 18.5 male offspring exposed to a maternal high-fat low-fiber diet, *G. vaginalis* 11E4 vaginal colonization, or a combination (linear fit model, FDR < 0.1, log(FC) = 1.5). Color based on row *Z*-scores for each gene. Five clusters of differentially regulated genes in each treatment group are indicated. *N* = 3 males per treatment. **F** Survival of offspring from dams that experience a single or multiple compounding adversities in a second validation cohort. All pups were C-section delivered and gavaged with human CST IV inoculant, showing the highest offspring mortality risk in HFt-LFb+Gv male offspring exposed to CST IV at birth. LFt-HFb = low-fat high-fiber; HFt-LFb = high-fat low-fiber; Gv = *G. vaginalis* 11E4. Kaplan–Meier survival analysis. *N* = 24–40 offspring per treatment condition.

As the impact of postnatal microbial colonization on circulating neutrophils and offspring morbidity may be related to prenatal programmatic effects of maternal high-fat low-fiber diet and presence of vaginal *G. vaginalis*, we next examined the transcriptional landscape of the placenta and ileum from E18.5 male fetuses. We repeated the two-hit model experiment in an independent validation cohort, and replicated excessive weight gain, delayed glucose clearance, and increased circulating glucose levels in females following consumption of a high-fat low-fiber for six weeks (Supplementary Fig. 12a–c). Gene expression patterns of the placenta and ileum were analyzed in embryonic day 18.5 male offspring from LFt-HFb dams, male offspring from LFt-HFb dams colonized with *G. vaginalis* (LFt-HFb+Gv), male offspring from HFt-LFb dams (HFt-LFb), and male offspring from HFt-LFb dams colonized with *G. vaginalis* (HFt-LFb+Gv) by bulk RNA sequencing (Fig. 4A).

In the placenta, differential gene expression analysis (FDR < 0.01, log(FC) = 1.5) revealed that nearly one-third, 110 of 351, of genes with significant differences were altered in the HFt-LFb male placenta compared with LFt-HFb male placenta, with the majority of these genes downregulated in HFt-LFb relative to LFt-HFb male placentas (Fig. 4B,C). Functional enrichment of these HFt-LFb-specific downregulated genes demonstrated enrichment in pathways involved in the response to changing nutrient levels, fatty acid metabolism, dopamine transport, and serotonin transport. Additionally, 173 of 351 genes were differentially expressed between HFt-LFb+Gv and LFt-HFb male placenta. Functional enrichment of these differentially expressed genes showed enrichment for pathways involved in collagen degradation, response to inflammation, and receptor-mediated endocytosis (Supplementary Fig. 13a).

Geneset enrichment analysis (GSEA) was next used to identify pathways that were altered by *G. vaginalis* 11E4 vaginal colonization, a high-fat low-fiber diet, or in combination. Comparison of genesets between LFt-HFb+Gv and LFt-HFb male placentas showed significant enrichment of pathways involved in the activation of innate immune responses, interleukin-6 biosynthetic processes, and negative regulation of interleukin-10 production in the LFt-HFb+Gv placenta compared to male placenta from LFt-HFb dams (Supplementary Fig. 14a). Male placenta samples from HFt-LFb dams showed significant disruption in pathways involved metabolic processes, including steroid metabolic processes and oxidative phosphorylation compared with LFt-HFb male placentas (Supplementary Fig. 14b). Further, comparison between HFt-LFb+Gv and HFt-LFb male placentas revealed significant disruption in adaptive immune response pathways in HFt-LFb+Gv placentas, pointing to a transcriptional signature of an impaired immune response in the HFt-LFb male placenta following maternal vaginal exposure to *G. vaginalis* (Supplementary Fig. 14c). Consistent with this notion of impaired maturational processes, comparisons between HFt-LFb+Gv and LFt-HFb males revealed significant disruption to pathways involved in the regulation of circulatory processes and regulation of vasculature development in the placenta of HFt-LFb+Gv males relative to that of LFt-HFb males (Supplementary Fig. 14d).

As alterations to placental handling of nutrients, metabolites, and oxygen transport influences the development of fetal tissues^83^, we used bulk RNA sequencing to determine the possible compounding effect of maternal diet and *G. vaginalis* vaginal colonization on the fetal ileum. Differential gene expression analysis (FDR < 0.1, log(FC) = 1.5) revealed that there were 285 of the total 781 differentially expressed genes in the fetal ileum of LFt-HFb and LFt-HFb+Gv males (Fig. 4D,E). Additionally, 232 of 781 genes were differentially expressed between HFt-LFb+Gv and LFt-HFb male ileum. We also found a cluster of genes that were downregulated in the ileum of HFt-LFb+Gv males relative to LFt-HFb, LFt-HFb+Gv, and HFt-LFb males (Supplementary Fig. 13b). These downregulated genes were enriched in pathways involved in oxygen transport, antigen processing, cholesterol metabolic processes, lymph node development, heme metabolic process, and regulation leukocyte migration, suggesting disruption of important maturational processes in the ileum that only occurred due to combinatorial exposure to maternal HFt-LFb and *G. vaginalis* vaginal colonization.

Paralleling the broad disruption to gene pathways involved in metabolic and immune signaling cascades in the placenta, GSEA showed significant alterations in the fetal ileum due to maternal diet, infection, and their combination. Comparison of genesets showed significant enrichment of pathways involved in immune activation and acute inflammatory responses in the LFt-HFb+Gv male fetal ileum compared with LFt-HFb male ileum (Supplementary Fig. 15a). Male ileum samples from HFt-LFb dams showed significant disruption in maturational pathways, including epithelial cell development (Supplementary Fig. 15b). Further, comparison between HFt-LFb+Gv and HFt-LFb male ileum revealed significant differences in genesets involved the regulation of vasculature development, epithelial cell proliferation, and myeloid mediated immunity in HFt-LFb+Gv ileum, consistent with disruption to transcriptional processes involved in the maturation of the ileum (Supplementary Fig. 15c). Collectively, these results showed that presence of vaginal *G. vaginalis* and maternal consumption of a HFt-LFb diet during pregnancy resulted in broad transcriptional dysregulation in the placenta and ileum that was not observed in either treatment alone. These difference in gene expression patterns in the HFt-LFb+Gv placenta and ileum may reflect alterations to transcriptional pathways that may support critical maturational and developmental processes and warrants further investigation. Lastly, to directly link these transcriptional alterations with offspring mortality risk, a separate cohort of offspring from all treatment groups were C-section delivered and inoculated with CST IV. Replicating the results from our discovery cohort, HFt-LFb+Gv offspring colonized with CST IV showed the highest mortality rate when compared with LFt-HFb, HFt-LFb, LFt-HFb+Gv male offspring exposure to CST IV (Fig. 4F). Collectively, our results suggest that maternal diet and the vaginal microbiota impact gene expression patterns in fetal tissues, and that, in turn, may modulate the postnatal response to the colonizing microbiota in newborn mice.

## Discussion

The transition from intrauterine to extrauterine environments involves exposure to maternal microbial communities. The composition of these pioneer communities shows a high degree of inter-individual variability, but the extent to which this heterogeneity may be linked to long-term health outcomes is incompletely understood. To begin to address these complex interactions, we first determined whether vertical transmission of distinct human vaginal microbial communities contributed to lasting offspring outcomes. We used our previously developed paradigm of cesarean delivery of embryonic day 18.5 mice, to prevent birth-associated colonization by murine microbiota, and then inoculated the neonates by oral gavage with distinct human vaginal microbial communities obtained from women late in pregnancy^44^. Our comparisons focused on the *Lactobacillus crispatus*-dominant community state type (CST I) and the community state type that lacks *Lactobacillus* dominance and is replaced by anaerobic bacteria, including *G. vaginalis* (CST IV)^22,24,36,37^. These microbiota exhibit community-specific metabolic and immunologic effects in the female reproductive tract, enabling us to examine how vertical transmission of distinct maternal microbial communities may have lasting impacts on growth, immunity, and brain development.

Application of this mouse model revealed that birth-associated exposure to CST I and CST IV produced a male-specific effect on postnatal body weight trajectories, where CST IV males showed a significant increase in body weight. We further observed shifts in circulating immune composition and changes to gene expression patterns in the paraventricular nucleus of the hypothalamus in CST IV adult males, which may reflect altered feeding patterns and metabolic regulation that could manifest in changes to growth trajectories and body weight observe postnatally. These lasting vaginal community state type-specific effects on offspring outcomes pointed to possible programmatic effects of these microbiota early in life. The neonate intestinal tract exhibits a number of transcriptional, metabolic, and immune adaptations that balance an appropriate immune response to the colonizing microbiota while also minimizing the overt immunopathology that may result from the colonization response^9,84–86^. Rodent models have shown that activation of these processes is delayed in cesarean delivered mice and missing this window of opportunity is associated with increased susceptibility to infection across the lifespan^87,88^. Additionally, our results suggest that colonization by CST I and CST IV may stimulate comparable CST-specific pathways in the postnatal ileum in mouse offspring as has been observed in the female reproductive tract^60,61,89^. Surprisingly, our studies showed that vaginally delivered and CST IV inoculated offspring were more similar on various endpoints relative to CST I and noninoculated C-section delivered offspring, including body weight and gene expression patterns in the neonatal ileum and adult hypothalamus. One possible explanation relates to the immunogenic potential of CST I, CST IV, and the Gammaproteobacteria*-*enriched community that colonize vaginally delivered mice. For instance, in vitro colonization of fetal enterocytes with *Lactobacillus* induces a slow and more sustained immune response while colonization by the Gammaproteobacteria *E*. *coli* more rapid immune signaling cascades in fetal enterocytes^90^. This may suggest that the observed differences in gene pathways involved in mucus secretion, defense response, and leukocyte recruitment in the ileum of CST IV and vaginally delivered males compared with CST I males may be related to the threshold of induction between these two communities, but this premise requires formal testing.

Recent advances in high-dimensional, systems-level analyses have permitted longitudinal monitoring in response to the rapidly changing microbial landscape across development^91^. Applying a similar protocol to our mouse model revealed that neutrophils constitute the largest fraction of the circulating immune compartment of 1-day-old CST I, CST IV, and VD males. Deeper analysis revealed presence of CD14+ subcluster of neutrophils, consistent with a recent study showing that the transitory accumulation of these immune cells shows suppressive activity and may be critical for regulating inflammation in the newborn^92,93^. While it is tempting to speculate on the functional connection between colonization by distinct maternal microbiota and CD14+ neutrophils, additional work using single-cell immunophenotyping will be needed to assess the functional importance of the heterogeneity of circulating neutrophils during the first 72 h of postnatal life. Our observation of colonization-dependent decrease in circulating basophils is consistent with previous work demonstrating that germ-free mice had increased steady-state basophil populations and increased allergic inflammation, while the presence of an intact microbiome limits IgE levels, decreases circulating basophil populations, and lowers risk for allergic inflammation^94^. The possible relationship between colonization by distinct microbial communities, basophils and lasting health outcomes is an important avenue for future research. Further, differences in the frequency of CD4^+^ T cells between vaginally delivered males and cesarean delivered males may highlight important contributions of mode of delivery and developmental maturity to circulating T cell populations that warrants further examination^91^. A potential limitation of this current model is the reliance on specific pathogen free mice, whereby offspring are subsequently colonized by microbiota specific to the foster dam. Utilizing germ-free mice may provide an additional avenue for resolving key insights into the functional contribution of CST-specific exposure at birth in a manner that is independent of subsequent waves of colonization. Indeed, as the human vaginal microbiota transiently colonizes the infant gut and is replaced by more stably colonizing microbiota from the maternal gut, additional studies are now possible to examine the causes and consequences of subsequent colonization by maternal human skin, gut, and breastmilk-associated microbiota in this model^3,95^. The methods reported here provide additional avenues to using a murine model to examine mechanistic roles of human microbial communities on immune system maturation, metabolic processes, brain development, and how these early-life events may influence health outcomes later in life.

It is well established that maternal environmental exposures during pregnancy, such as infection or malnutrition^7^, impact prenatal development and increase offspring vulnerability to subsequent environmental stressors across the lifespan^39,65–67^. Consumption of a high-fat low-fiber diet increased maternal body weight, delayed glucose clearance, and altered gut microbiota composition, paralleling previous studies using female mice. Interestingly, gut microbiota composition did not converge at any point during pregnancy between LFt-HFb and HFt-LFb dams, emphasizing the need to better understand the role of prepregnancy gut microbiota structure and function in pregnancy-related outcomes as an important avenue for future research.

In examining the prenatal programmatic effects of being exposed to a maternal high-fat low-fiber diet and vaginal colonization of *G. vaginalis*, gene expression analyses of the placenta and fetal ileum identified differences in gene pathways involved in tissue maturation that were detected only when modeling multiple maternal risk factors in parallel. These tissue-specific alterations in gene expression patterns were associated with an excessive influx of neutrophils following intestinal colonization by human vaginal microbiota at birth. This heightened innate immune response may be associated with a differential risk in offspring morbidity. A similar immune response to colonizing bacteria has been reported in premature infants and is associated with the breakdown of the mucosal barrier, bacterial translocation, and increased risk for devastating neonatal diseases such as necrotizing enterocolitis^96^. In this light, our results may implicate the involvement of maternal risk factors, such as diet-induced obesity and the presence of a nonoptimal vaginal microbiota, in shaping the postnatal response to microbiota from maternal and environmental depots, and further highlight the importance of better understanding the role of compounding maternal risk factors in adverse neonatal outcomes.

Certainly, additional work is required to identify the mechanisms by which the maternal prenatal environment and microbiota-derived signals interact to contribute to adverse neonatal outcomes and produce lasting effects across the lifespan. Our findings highlight that compounding risk factors, such as chronic consumption of a high-fat diet and presence of a nonoptimal vaginal microbiota, may negatively impact key aspects of prenatal development with important implications for offspring health outcomes in the postnatal environment. Translationally, our work emphasizes the potential importance of collecting information on maternal exposures in ongoing clinical trials assessing the utility of seeding cesarean delivered infants with maternal cervicovaginal and fecal microbiota. Inclusion of maternal exposure history may provide insight into which classes of microbiota may or may not exhibit therapeutic value for the newborn. Further, given the modifiable nature of the microbiota composition and function, as highlighted by the recent implementation of microbiota-directed food interventions^97,98^, our preclinical study underscores the importance of additional work focused on interventions that target maternal microbial composition and function to promote optimal maternal and fetal health.

## Methods

### Human samples

Human vaginal secretions were selected from a collection of vaginal swabs self-collected weekly until delivery by pregnant women enrolled in the Birth, Eating, and the Microbiome (BEAM) study at the University of Maryland Baltimore. The clinical study protocol (HP-00056389) was approved by the Institutional Review Board of the University of Maryland Baltimore. Written informed consent was appropriately obtained from all participants. Samples were self-collected using the Copan ESwab system and stored frozen at −20 °C in 1 ml of Amies Transport Medium to preserve vaginal microbiota composition until transport to the laboratory where the samples were stored at −80 °C. As part of the parent study the vaginal microbiota composition of all samples collected was established using previously validated protocols^99^, CST were established using VALENCIA, a nearest centroid classification method for vaginal microbial communities based on composition^20^. Sample selection criteria for transplantation experiments included: (1) harboring a CST I or CST IV vaginal microbiota, (2) availability of samples between 36 and 39 weeks of pregnancy, and (3) women remained in the respective CST throughout pregnancy. The microbiota seeded in Amies transport medium was used as the inoculant (20 µL).

### Animals

All experiments were approved by the University of Maryland School of Medicine Institutional Animal Care and Use Committee and performed in accordance with National Institutes of Health Animal Care and Use Guidelines (Protocol #0517001). Mice were housed under a 12 h light/day photoperiod with lights on an 0700 EST and ad libitum access to water and a grain-based chow diet (Purina Rodent Chow, St. Louis, MO; 28.1% protein, 59.8% carbohydrate, 12.1% fat). Additional details on diet manipulation experiments appear below. Timed pregnancies were established by introducing a breeder male to a cage housing two females within one hour prior to lights off. Males were removed and copulation plugs were checked within one hour of “lights on” to estimate the number of animals that were going to be used in the study. Noon on the day that the plug was observed was considered embryonic day 0.5 (E0.5). Samples from one male and one female per litter were used for all subsequent analyses.

### Cesarean delivery surgery

C-section surgeries were conducted as previously described in a series of validation experiments, in which C-section delivered pups were inoculated with mouse vaginal microbiota and now extended to investigate the role human vaginal microbiota^44^. Donor mice used in these studies were C57Bl/6 J females ordered from JAX laboratories at 4 weeks old. Foster mice used in these studies were on a C57BL/6:129 background that are maintained in-house as an outbred mixed colony. This mixed colony is used as the foster based on well-documented occurrence of infanticide of nongenetically related offspring in C57Bl/6 J dams^47^. In published and current studies using mixed colony females has yielded a 100% acceptance rate of foster animals^44^. Foster females were time-mated 24 h before the expected surgery of donor females (E18.5). Foster females delivered a day prior to donor to ensure successful acceptance of the fostered litter. Cesarean deliveries of offspring were conducted under sterile conditions, and environmental swabs were taken from surgery suite area to account for contamination. For the surgery, immediately following rapid decapitation, dams were submerged in 70% EtOH for 30 s to minimize contamination of the uterine horn from maternal skin bacteria during extraction. Following removal, uterine horns were placed on a sterile field inside a sterile incubator. Once the fetus and placenta were excised from the uterine horn, the placenta and membranes that surround pups were removed and the umbilical cord was ligated. The remaining membranes and fluid from nose and mouth of the pup were cleared, and after breathing was stimulated, pups were kept warm on a sterile field inside a separate sterile incubator (Rodent Warmer ×1, Braintree Scientific Inc., MA). Offspring with overt signs of developmental delay, defined as small body size relative to litters or presence of a neighboring resorption site, were excluded from experiments. Time from maternal euthanasia to stimulation of offspring breathing was 2 min across all experiments. All pups were checked for viability prior to microbial transplantation by observance of healthy skin color and toe pinch withdrawal reflex. Time from stimulation of breathing to transplantation of human microbiota was 10 minutes.

### Mouse microbial colonization

The microbial colonization and fostering procedures were conducted using our previously validated approach^33^. On the day of colonization experiments, 20 µL of selected human vaginal secretion samples stored in Amies transport medium were retrieved from the −80 °C and thawed on ice. Upon thawing, samples were briefly centrifuged to ensure any residual liquid was pelleted. Samples remained on ice during surgeries. C-section pups were randomly assigned to be inoculated with Amies transport medium only (Amies), CST I or CST IV samples. Vaginally delivered offspring were born within 0.5 days of C-section delivered pups. Orogastric gavage occurred by guiding a silastic catheter (Intramedic Polyethylene Tubing PE 10, Becton Dickinson, Franklin Lakes, NJ) into the neonate’s esophagus, upon which offspring were fed once with 20 µL of fluid containing human vaginal microbiota or Amies only. Pup vital signs were checked at this time. Concurrently, a foster dam was removed from her cage and placed into an opaque container.The pups born to foster dams were euthanized at this time. One male and one female from each treatment group (Amies, CST I, CST IV and vaginally delivered) were represented within each litter transferred to the empty foster dam cage. Age-matched vaginally delivered offspring consisted of pups birthed by a treatment naïve donor dam that were cross-fostered to a foster dam at the same time as C-section offspring. Upon transfer of all foster pups, the foster dam was reintroduced to her cage, and the cage was returned to the colony room. Time elapsed from C-section, inoculation, and transfer to foster dam was 30 min.

All animals were checked daily following surgery for the presence of a milk dot, indicating successful feeding and acceptance of pup by the foster mother. Pups were checked every 12 h for the first 5 days, after which point pups were checked weekly. Offspring remained in these cages until weaning, upon which offspring were weaned into same-sex cages and remained in these cages until tissue collections. This protocol was independently repeated five times, for a total of five technical replicates. For statistical analysis, the individual offspring constituted the data point in these experiments, and this is reflected in figure legends.

### Validation of human vaginal microbiota in mouse gastrointestinal tract

Twenty-four and 48 h following inoculation, offspring were euthanized, the entire gastrointestinal tract was excised, and the milk dot, ileum, jejunum, ileum and cecum, and colon samples were individually segmented and collected for 16 S rRNA gene sequencing analysis, as we have done previously (Jasarevic et al., 2018, Nature Neuroscience). Samples were rapidly frozen in liquid nitrogen and stored at −80 °C until DNA extraction. All samples were processed as described in the “Genomic DNA isolation and 16 S rRNA marker gene sequencing” section.

### Early-life microbiota source tracking

To examine the possible origins of microbiota present in the postnatal intestinal tract of C-section delivered offspring, we collected samples from the local cage environment and the foster dam. To account for microbial density in the cage environment, foster dam cages were not changed within one week of C-section cross-fostering experiments. Local cage environment samples included corn cob that surrounded the maternal nest, and the cotton bedding with which the pup litter had direct contact. Dried fecal pellets were removed from both environmental samples to ensure capturing sample-specific microbial community composition. Maternal samples included maternal oral, skin swabs of the nipple chain, cecal, colon, and vaginal. All samples were processed as described in “Genomic DNA isolation and 16 S rRNA marker gene sequencing.”

### Assessments in C-section offspring

For offspring, weekly body weights were collected starting at postnatal day 7 and fecal pellets were collected starting at postnatal day 21 to examine the impact early-life colonization on growth and microbiota maturation. On postnatal day 56, male and female adults were sacrificed, and body measurements, intestinal segments, blood, and brain samples were collected. Tissues were processed as described below.

### RNA-seq analysis of the adult paraventricular nucleus of the hypothalamus (PVN)

Frozen brains from postnatal day 56 males were cryosectioned at −20 °C. Using a hollow 1.0 mm needle, the PVN was removed according to the mouse brain atlas^100^. PVN micropunches were immediately dispensed into 500 µl of Trizol and stored at −80 °C until processing. Messenger RNA was purified RNeasy Mini kit (Qiagen). Illumina single-end cDNA libraries of adult male PVN mRNA were prepared from 250 ng total RNA using the TruSeq Stranded mRNA Kit with poly-A enrichment (RS-122-2101, Illumina) according to the manufacturer’s protocol. Samples were multiplexed and sequenced on two identical NextSeq500 lanes using high-output 1 × 75 bp chemistry (Illumina). The concatenated FASTQ files generated from Illumina were used as input to kallisto^101^, a program that pseudoaligns high-throughput sequencing reads to the *Mus musculus* reference transcriptome (version 38) and quantifies transcript expression. We used 60 bootstrap samples to ensure accurate transcript quantification. Gene isoforms were collapsed to gene symbols using the Bioconductor package tximport (version 3.4)^102^. Genes were filtered to counts per million >1 in at least three samples. The filtered gene list was normalized using trimmed mean of M-values in edgeR^103^. Variance weights were calculated using voom^104^, and differential expression analysis of linear fit models was performed using limma v3.12.3^105^.

### Maternal antibiotic exposure

To determine the influence of maternal microbial exposure on circulating immune cell composition in postnatal day 1 mice, pregnant donor dams were provided with ad libitum access to drinking water mixed with ampicillin, vancomycin and neomycin (all 1 mg/ml), and supplemented with 1.125 g aspartame to increase palatability, starting on gestational day 15^64^. On embryonic day 18.5 offspring were delivered by C-section and transferred to a foster dam, as described above. Whole blood was collected at postnatal day 1 for blood lymphocyte counts.

### Blood lymphocyte counts

Blood (30 µl) was collected from postnatal day 1 male offspring into EDTA-loaded tubes, and lymphocyte absolute counts and frequencies in the whole blood were determined using a Heska Element HT5 Veterinary Analyzer at the ZooQuatic Laboratory within the Institute of Marine and Environmental Technology.

### Postnatal day 1 ileum collections

On postnatal day 1 offspring were euthanized, the entire gastrointestinal tract was excised, and the ileum was rapidly frozen in liquid nitrogen and maintained in –80 °C until RNA isolation. For bulk RNA sequencing analysis, tissues were homogenized in Trizol, and messenger RNA was purified using the RNeasy Mini kit (Qiagen). Illumina single-end cDNA libraries of postnatal day 1 ileum mRNA was prepared from 250 ng total RNA using the TruSeq Stranded mRNA Kit with poly-A enrichment (RS-122-2101, Illumina) according to the manufacturer’s protocol. Library fragment size was quantified using an Agilent High Sensitivity D1000 ScreenTape Assay on an Agilent 4200 Tapestation System (G2991AA). Library concentration was quantified using the Qubit dsDNA HS (High Sensitivity) Assay Kit. Samples representative of all treatment groups were multiplexed and sequenced on an Illumina NextSeq500 instrument using high-output 1 × 75 bp geometry (Illumina).

### High-fat low-fiber dietary manipulation

A prepregnancy diet-induced obesity approach was used to determine how maternal exposures influence offspring response to the colonizing microbiome at birth. A separate cohort of postnatal day 28 C57Bl/6 J females purchased from JAX were allowed to acclimate for 2 weeks prior to investigation. During this acclimation period, all females consumed a low-fat high-fiber (LFt-HFb) diet (grain-based chow, Purina 5001). Following acclimation, females were randomly assigned to the high-fat low-fiber diet (HFt-LFb; ResearchDiets Inc, D12492) or remained on the low-fat high-fiber (LFt-HFb) diet (grain-based chow, Purina 5001). Following acclimation, females were randomly assigned to the high-fat low-fiber diet (HFt-LFb; ResearchDiets Inc, D12492) or remained on the low-fat high-fiber (LFt-HFb) diet (grain-based chow, Purina 5001). Females had ad libitum access to respective diets for the span of the experiment. An important note on treatment group notation: For these experiments we explicitly highlight the low soluble fiber in commercially available refined high-fat diet formulations due to accumulating evidence that the lack of soluble fiber in these dietary formulations is an important contributor to excessive weight gain, obesity, and diabetes in mouse models of diet-induced metabolic syndrome and obesity^106,107^.

### Glucose tolerance test

To confirm a prediabetic state in HFt-LFb females, glucose tolerance test was administered in 12-week-old females following six weeks on either HFt-LFb or LFt-HFb diets. Food was removed at 0800 EST and mice were fasted for 6 h, upon which mice were injected intraperitoneally with 0.3 g/mL glucose in saline. Glucose readings were collected by tail blood at 0-, 30-, 60-, and 120 min timepoints using the Contour Next Blood Glucose Monitoring System (Bayer Co, Germany). Pilot experiments revealed that ~15% of females consuming the HFt-LFb diet were resistant to diet-induced body weight gain and intolerance. As a result, we established the following inclusion criteria: females on the respective diets do not overlap by two standard deviations on body weight and glucose tolerance test were excluded from experiment before breeding and subsequent treatments. Females remained on the assigned diets for the span of the experiment. Females were mated following confirmation of pregestational body weight and glucose tolerance. For the discovery cohort, 15 LFt-HFb and 20 HFt-LFb females were used. Due to increased variability in body weight gain and glucose tolerance response that we observed in the discovery cohort, 20 LFt-HFb and 50 HFt-LFb females were used for the validation cohort.

### Serial tracking and analysis of maternal fecal microbiota before and during pregnancy

To determine the impact of chronic depletion of soluble fiber on the background of a high-fat diet on gut microbiota prior and during pregnancy, a serial sampling strategy was used to examine the impact of dietary transition, chronic HFt-LFb diet consumption, and impact of HFt-LFb diet of pregnancy-associated gut microbiota dynamics, an approach we have previously used to study the effects of stress during pregnancy on the maternal gut microbiota. Fecal pellets were collected prior to transition to HFt-LFb diet, weekly fecal samples were collected during consumption of either HFt-LFb or LFt-HFb diet, and upon confirmation of copulation plug, daily fecal samples were collected during pregnancy.

### Quantitative PCR of adult female intestinal segments

Following body weight changes and glucose tolerance test, a subcohort of females were euthanized, intestinal segments were collected, rapidly frozen in liquid nitrogen, and stored at −80 °C. Tissues were homogenized in Trizol, and messenger RNA was purified using the RNeasy Mini kit (Qiagen). Transcript counts of hypoxia-inducible factor-1-alpha (Mm00468869_m1) was assayed by TaqMan gene expression assay (ThermoFisher Scientific).

### Cultivation and culture of CST IV strain *Gardnerella vaginalis* 11E4

The cultivation-dependent confirmation of CST I and CST IV is a routine assay conducted by the Ravel group to ensure that the vaginal fluid samples deposited into the liquid Amies can be harvested. *Gardnerella vaginalis* isolate 11E4 were maintained on Human Bilayer Tween Agar (BD) plates and NYC III medium according to the manufacturer’s instructions. Agar plates and liquid cultures were incubated at 37 °C with anaerobic gas mixture, 80% N_2_, 10% CO_2_, and 10% H_2_. Fresh live cultures were used for inoculation experiments and density was confirmed before each inoculation by dilution plating and counting colony forming units (CFU).

### Midgestational colonization by *Gardnerella vaginalis* strain 11E4 to model presence of a common member of CST IV

To model the local and peripheral inflammation observed in the presence of *G. vaginalis* in the cervicovaginal space of pregnant women in a clinical setting, pregnant dams from the dietary manipulation experiments were inoculated intravaginally with 50 µL of 2 × 10^8^ CFU/ml saline of *G. vaginalis* strain 11E4 at gestational day 13.5 and E15.5, similar to previous studies^77,79^. *G. vaginalis* strain 11E4 was isolated from a women with BV (Nugent Score 7), a vaginal pH of 4.7, and a CST IV vaginal microbiota (subject 15 in ref. ^23^). The strain carries all known pathogenic traits associated with *G. vaginalis* proper, including the expression of vaginolysin, a cholesterol-dependent pore-forming cytolysins implicated in vaginal dysbiosis^108,109^. This strain was selected based on pilot testing conducted by the Ravel lab in which 14 strains of human *G. vaginalis* were evaluated for biofilm formation, a feature associated with symptomatic bacterial vaginosis^108,109^. Based on this, we tested two biofilm forming strains (11E4 and 16B1) and confirmed the presence of *G. vaginalis* 48 h post-inoculation in mouse vaginal fluid by qPCR for *rpoB*. Days of inoculation (E13.5, E15.5) were selected based on previous work showing that introduction of *G. vaginalis* on these days induces an inflammatory response without significantly impacting placentation or inducing delivery. Pilot experiments revealed that 11E4 was more readily present in the mouse vaginal lavage fluid than 16B1, and thus only used 11E4 for the present experiments. Live *G. vaginalis* cultures were inoculated at each timepoint, and colony forming unit counts were performed on Human Bilayer Tween agar for each batch of *G. vaginalis* cultures to ensure that females are inoculated with similar densities of pure live *G. vaginalis* cultures that were free of contamination.

### Genomic DNA isolation and 16 S rRNA marker gene sequencing

Genomic DNA from fecal pellet samples were isolated using the Stratec PSP Spin Stool DNA Plus kit using the difficult to lyse bacteria protocol from the manufacturer (STRATEC Molecular GmbH, Berlin, Germany). Each sample DNA was eluted into 100 µL of Elution Buffer provided by the Stratec PSP Spin Stool DNA Plus kit. The V4 region of the bacterial 16 S rRNA gene was amplified using a dual-index paired-end sequencing strategy for the Illumina platform as previously described^45^. Sequencing was performed on a MiSeq instrument (Illumina, San Diego, CA) using 2 × 250 base paired-end chemistry at the University of Maryland School of Medicine Institute for Genome Sciences. The sequences were demultiplexed using the dual-barcode strategy, a mapping file linking barcode to samples and split_libraries.py, a QIIME-dependent script. The resulting forward and reverse fastq files were split by sample using the QIIME-dependent script split_sequence_file_on_sample_ids.py, and primer sequences were removed using TagCleaner (version 0.16). Further processing followed the DADA2 workflow for Big Data and DADA2 (v.1.5.2) (https://benjjneb.github.io/dada2/bigdata.html). Data filtering was set to include features where 20% of its values contain a minimum of four counts. In addition, features that exhibit low variance across treatment conditions are unlikely to be associated with treatment conditions, and therefore variance was measured by interquartile range and removed at 10%. Data were normalized by cumulative sum scaling and differential abundance analysis was conducted using Linear Discriminant Analysis effect size with an FDR cutoff at q < 0.05. For quality control purposes, water and processed blank samples were sequenced and analyzed through the bioinformatics pipeline. Taxa identified as cyanobacteria or ‘unclassified’ to the phylum level were removed. For confirmation of human vaginal microbiota, taxonomic assignments were performed using a combination of a phylogenetics-based classifier and speciateIT software (http://www.speciateIT.sourceforge.net).

### Fetal tissue analysis

Fetal placenta and ileum samples were collected to examine the effect of the in utero environment shaped by maternal diet and the presence of a nonoptimal vaginal microbiome on gene expression patterns in these tissues. On E18.5, pregnant dams were rapidly decapitated by cervical dislocation. Litter characteristics, such as intrauterine position, number of offspring, sex ratio, and resorption sites, were noted. Fetal intestinal tracts and placenta samples were rapidly frozen in liquid nitrogen and maintained in −80 °C until RNA isolation. To control for the significant contribution of uterine horn laterality and intrauterine position, we selected fetal intestinal tract samples from conceptuses that were required to be from the first third of the embryos from cervical end; male samples flanked by two females (2 F males) were excluded, and embryos could not exhibit overt signs of developmental delay (for example, small size relative to litters and no presence of a neighboring resorption site). Tail snip from embryos were collected and retained for determination of sex by genotyping using primers specific to Jarid1 (5′-TGAAGCTTTTGGCTTTGAG-3′ and 5′-CCGCTGCCAAATTCTTTGG-3′). Reactions were incubated at 94 °C for 5 min, followed by 35 cycles of 94 °C for 20 s, 54 °C for 1 min, and 72 °C for 40 s, followed by 72 °C for 10 min. Following thermal cycling, 15 µL from each PCR product were mixed with 5 µL 6× loading dye solution (Sigma) before being loaded onto a 2% (w/v) agarose gel (Sigma) next to 0.5 µg 100 bp DNA ladder (Sigma) and electrophoresed in lx TAE buffer (40 mM Tris-acetate, 2 mM EDTA) at 120 V/cm for 45 min.

For bulk RNA sequencing analysis, tissues were homogenized in Trizol, and messenger RNA was purified using the RNeasy Mini kit (Qiagen). Illumina single-end cDNA libraries of E18.5 fetal intestinal tract and placenta mRNA was prepared from 250 ng total RNA using the TruSeq Stranded mRNA Kit with poly-A enrichment (RS-122-2101, Illumina) according to the manufacturer’s protocol. Library fragment size was quantified using an Agilent High Sensitivity D1000 ScreenTape Assay on an Agilent 4200 Tapestation System (G2991AA). Library concentration was quantified using the Qubit dsDNA HS (High Sensitivity) Assay Kit. Samples representative of all treatment groups were multiplexed and sequenced on an Illumina NextSeq500 instrument using high-output 1×75-bp geometry (Illumina).

### Quantitative analysis of vaginal bacteria by qPCR

Genomic DNA from vaginal fluid recovered in 50 µL saline were isolated using the Stratec PSP Spin Stool DNA Plus kit using the difficult to lyse bacteria protocol from the manufacturer (STRATEC Molecular GmbH, Berlin, Germany). Each sample DNA was eluted into 100 µL of Elution Buffer provided by the Stratec PSP Spin Stool DNA Plus kit. Gene copy number of *G. vaginalis rpoB* was determined by quantitative real-time PCR analysis, performed on an Applied Biosystems QuantStudio6 Flex Real Time PCR System (Life Technologies, Grand Island, NY). Genomic DNA was extracted using Stratec PSP Spin Stool DNA Plus kit from vaginal swabs, TaqMan Assays specific for *G. vaginalis rpoB*, panbacterial 16 S rRNA genes, TaqMan Vaginal Microbiota Amplification Control, and TaqMan Fast Advanced Master Mix (Life Technologies). Gene copy number counts were calculated using a standard curve method and the 16 S rRNA gene level as an internal standard.

### Whole blood sample collection and storage

Thirty microliters whole blood from postnatal day 1 and 100 µL whole blood from postnatal day 56 mice was collect for mass cytometry analysis and mixed with Cytodelics Whole Blood Cell Stabilizer at a 1:1 ratio, incubated at room temperature for 10 minutes and transferred to a −80 °C freezer for long-term storage awaiting analysis. Whole blood samples preserved in Cytodelics stabilized were thawed at 20 °C for 5 min. Samples were then diluted 1:4 with Cytodelics Wash Buffer #1 and allowed to lyse red blood cells for 15 min. Cells were then washed twice with Cytodelics Wash Buffer #2, filtered through a 35-micron mesh, cells were counted on a hemacytometer, and immediately proceeded with barcoding and staining. In these experiments, we took advantage of a palladium-based barcoding approach (see, “Mass Cytometry Barcoding, Staining and Acquisition”) to limit the batch-to-batch variability that is inherent to single-tube samples. One limitation to this barcoding approach is the sample size per treatment group that can be included per run, and thus may present potential limitation in the interpretation of the CyTOF experiments. Additionally, application of correction for multiple comparison (i.e., Tukey’s post-hoc) was used to confirm robustness of statistical significance.

### Antibody labeling and custom conjugation

Purified monoclonal antibodies were either purchased preconjugated from Fluidigm or purchased from BioLegend in Maxpar Ready Purified Antibody formulation and conjugated in-house using the MAXPAR X8 polymer conjugation kit (Fluidigm Inc.) according to manufacturer’s protocol. Antibody concentration before and after conjugation was measured by NanoDrop, antibodies were diluted in Stabilizer, and antibodies were titrated prior to use in the staining panel.

### Mass cytometry barcoding, staining, and acquisition

A maximum of 2 × 10^6^ cells per whole blood sample were barcoded using six combinations of palladium isotopes (^102^Pd, ^104^Pd, ^105^Pd, ^106^Pd, ^108^Pd, ^110^Pd) with the Palladium barcoding kit (Fluidigm). Cells were fixed for 10 min in 1 mL of Fix I Buffer at room temperature, followed by two washes in 1 mL of MaxPar Perm Buffer. Barcodes were resuspended in 100 µL Perm Buffer, transferred to each sample and incubated for 30 min at room temperatures. All samples were then washed twice with Cell Staining Buffer (CSB) and then pooled. Cells were suspended in 150 ml antibody cocktail per 10^7^ cells and incubated for 30 min at room temperature. Antibodies used are listed in Supplementary Table 1. All antibodies were used a 1:100 dilution, with 1 µl per antibody used for up to 3 × 10^6^ live cells in total staining volume of 100 µl. After staining, cells were washed twice with CSB followed by overnight incubation in Iridium-labeled DNA-intercalator in 4% formaldehyde for a final concentration of 0.125 mM at 4 °C. Following two washes in Cell Staining Buffer, cells were rapidly frozen cryoprotective medium consisting of 90% FBS/10% DMSO. Following freezing, samples were shipped overnight to the University of Virginia School of Medicine Flow Cytometry Facility. On the day of acquisition, cells were washed once in Call Acquisition Buffer, once in PBS and twice in milliQ H2O filtered through a 35 mm nylon mesh and counted. Cells were diluted in milliQ H2O containing 10% EQ Four Element Calibration Beads. Samples were acquired a CyTOF2 mass cytometers with a wide bore configuration, using noise reduction, event length limits of 10–150 pushes and a sigma value of 3. Cells were acquired at a flow rate of 0.045 ml/min.

### Mass cytometry preprocessing and gating

All FCS files were preprocessed using Gaussian discrimination parameters, following recommendations by Fluidigm. Following this preprocessing step, FCS files were uploaded to the Astrolabe Cytometry Platform (Astrolabe Diagnostics, Inc.) where transformation, debarcoding, cleaning, labeling, and unsupervised clustering was done. Data was transformed using arcsinh with a cofactor of 5. Experimental batches were debarcoded and individual samples were then labeled using the Ek’Balam algorithm, a hierarchy-based algorithm for labeling cell subsets which combines knowledge-based gating strategy with unsupervised clustering using the FlowSOM algorithm. Differential expression analysis to compare treatment effects was conducted within the Astrolabe Cytometry Platform^110^. Groups average t-SNE maps and unsupervised clustering using FlowSOM^111^ were generated in Cytobank.

### Quantification and statistical analysis

Statistical information including sample size, mean, and statistical significance values are indicated in the text or the figure legends. A variety of statistical analyses were applied, each one specifically appropriate for the data and hypothesis, using the R statistical environment. For standard metabolic endpoints, analysis of variance (ANOVA) testing with repeated-measures corrections and Tukey post-hoc tests were used, with significance at an adjusted *p* < 0.05. Processing of RNA-Seq data were conducted using standardized and published protocols. Cytobank and Astrolabe Diagnostics were used for analysis of CyTOF data using default settings. GraphPad Prism and Adobe Illustrator were utilized for generating figures. No custom script was used to analyze RNA sequencing or cytometric data.

### Reporting summary

Further information on research design is available in the Nature Research Reporting Summary linked to this article.

## Supplementary information

Supplementary Information

Reporting Summary

## Data Availability

The RNA-seq and microbiota data generated in this study have been deposited in the NCBI SRA database under accession code PRJNA768872. All other raw data are available upon request without restrictions. Source data are provided with this paper.

## Source data

Source Data File

## Code availability

No custom script was used to analyze data.
