# Supplementary material for: The composition of human vaginal microbiota transferred at birth affects offspring health in a mouse model
Source: Nat Commun. 2021 Nov 1;12:6289. doi: 10.1038/s41467-021-26634-9 (PMC8560944; doi:10.1038/s41467-021-26634-9)
Supplement: Supplementary file 1 — Supplementary Information [file 41467_2021_26634_MOESM1_ESM.pdf]

Supplementary Information for

The composition of human vaginal microbiota transferred at birth affects offspring health in a mouse model

Eldin Jašarević[1,2†], Elizabeth M. Hill[1,2], Patrick J. Kane[1,2], Lindsay Rutt[3,4], Trevonn Gyles[1,2], Lillian Folts[1,2], Kylie D. Rock[1,2], Christopher D. Howard[1,2], Kathleen E. Morrison[1,2], Jacques Ravel[3,4] and Tracy L. Bale[1,2,5*]

*Correspondence to: tbale@som.umaryland.edu

This file includes:

Supplementary Table: 1
Supplementary Figures and Legends: 1-15

Other supporting files:
Source Data File

Supplementary Table 1. Reagents and Resources used in this report

REAGENT or RESOURCE	SOURCE	IDENTIFIER
Antibodies		
CD45 (30-F11) – 89Y	Fluidigm	Cat. # 3089005B
Ly6G (1A8) – 141Pr	Fluidigm	Cat. # 3141008B
CD11c (N418) – 142Nd	Fluidigm	Cat. # 3142003B
TCRb (H57-597) – 143Nd	Fluidigm	Cat. # 3143010B
CD115 (AFS98) – 144Nd	Fluidigm	Cat. # 3144012B
CD4 (RM4-5) – 145Nd	Fluidigm	Cat. # 3145002B
CD11b (M1/70) – 148Nd	Fluidigm	Cat. # 3148003B
CD19 (6D5) – 149Sm	Fluidigm	Cat. # 3149002B
IgD (11-26c.2a) – 150Nd	Fluidigm	Cat. # 3150011B
IgM (RMM-1) – 151Eu	Fluidigm	Cat. # 3151006B
CD3 (145-2C11) – 152Sm	Fluidigm	Cat. # 3152004B
CD274/PD-L1 (10F.9G2) – 153Eu	Fluidigm	Cat. # 3153016B
CD27 (LG.3A10) – 154Sm	Fluidigm	Cat. # 3150017B
CD64 (X54-5/7.1) – 155Gd	Fluidigm	Cat. # 3151012B
CD14 (Sa14-2) – 156Gd	Fluidigm	Cat. # 3156009B
FoxP3 (FJK-16s) – 158Gd	Fluidigm	Cat. # 3158003A
F4/80 (BM8) – 159Tb	Fluidigm	Cat. # 3159009B
CD62L L-selectin (MEL-14) – 160Gd	Fluidigm	Cat. # 3160008B
CD90 (T24/31) – 161Dy	Fluidigm	Cat. # 3161009B
Ly6C (Hk1.4) – 162Dy	Fluidigm	Cat. # 3162014B
APC (APC003) – 163Dy	Fluidigm	Cat. # 3163001B
APC anti-mouse MerTK (2B10C42)	BioLegend	Cat. # 151508
CX3CR1 (SA011F11) – 164Dy	Fluidigm	Cat. # 3164023B
Biotin – 165Ho	Fluidigm	Cat. # 3165012B
Biotin anti-mouse CD192/CCR2 (SA203G11)	BioLegend	Cat. # 150628
CD117 cKit (2B8) – 166Er	Fluidigm	Cat. # 3166004B
CD8a (53-6.7) – 168Er	Fluidigm	Cat. # 3168003B
Ly6A/E (D7) – 169Tm	Fluidigm	Cat. # 3169015B
CD49b (HMa2) – 170Er	Fluidigm	Cat. # 3170008B
CD44 (IM7) – 171Yb	Fluidigm	Cat. # 3171003B
Maxpar Ready CD24 (M1/69)	BioLegend	Cat. # 101829
Maxpar Ready NK-1.1 (PK136)	BioLegend	Cat. # 108743
Maxpar Ready CD335/NKp46 (29A1.4)	BioLegend	Cat. # 137625
CD45R/B220 (RA3-6B2) – 175Lu	Fluidigm	Cat. # 3176002B
FceR1a (MAR-1) – 176Yb	Fluidigm	Cat. # 3176006B
I-A/I-E (M5/114.15.2) – 209Bi	Fluidigm	Cat. #3209006B
Bacterial and Virus Strains		
Gardnerella vaginalis strain 11E4	This paper	N/A
Biological Samples		

Human donor CST I (EM15_W1D7)	This paper	N/A
Human donor CST IV (UAB116_W1D7)	This paper	N/A
Human donor CST I (EM15_W1D7(2))	This paper	N/A
Human donor CST IV (UAB116_W1D7(2))	This paper	N/A
Human donor CST I Week 36 (BMR0017)	This paper	N/A
Human donor CST I Week 37 (BMR0017)	This paper	N/A
Human donor CST IV Week 36 (BMR0038)	This paper	N/A
Human donor CST IV Week 37 (BMR0038)	This paper	N/A
Human donor CST I Week 39 (BMR0089)	This paper	N/A
Human donor CST IV Week 38 (BMR0038)	This paper	N/A
Chemicals, Peptides, and Recombinant Proteins		
Vancomycin	Sigma-Aldrich	Cat. # V2002
Ampicillin	Sigma-Aldrich	Cat. # A9393
Neomycin	Sigma-Aldrich	Cat. # N6386
Sodium Azide	Sigma-Aldrich	Cat. # S2002
Bovine Serum Albumin	Sigma-Aldrich	Cat. # 05470
Fetal Bovine Serum	Sigma-Aldrich	Cat. # F2442
1X PBS	Lonza	Cat. # 04-409R
Maxpar Cell Staining Buffer	Fludigm	Cat. # 201068
Maxpar Water	Fluidigm	Cat. # 201069
Maxpar Fix I Buffer	Fluidigm	Cat. # 201065
Maxpar Fix and Perm Buffer	Fluidigm	Cat. # 201067
Cell-ID Intercalator-Ir—500µM	Fluidigm	Cat. # 201192B
Cell-ID Cisplatin	Fluidigm	Cat. # 201064
Maxpar X8 Multimetal Labeling Kit	Fluidigm	Cat. # 201300
Cell-ID 20-Plex Pd Barcoding Kit	Fluidigm	Cat. # 201060
EQ Four Element Calibration Beads	Fluidigm	Cat. # 201078
Sso Advanced Universal Probes Supermix	BioRad	Cat. # 1725280
Critical Commercial Assays		
RNeasy Mini Kit		
RNeasy Mini Kit	Qiagen	Cat. # 74104
TruSeq Stranded mRNA Kit	Illumina	Cat. # 20020595
Agilent High Sensitivity D1000 ScreenTape Assay	Agilent	Cat. # 5067-5584
Agilent High Sensitivity D5000 ScreenTape Assay	Agilent	Cat. # 5067-5592
Agilent High Sensitivity RNA ScreenTape Assay	Agilent	Cat. # 5067-5579
Qubit RNA HS Assay Kit	ThermoFisher	Cat. # Q32852
Qubit dsDNA HS Assay Kit	ThermoFisher	Cat. # Q32851
Stratec PSP Spin Stool DNA Plus kit	STRATEC Molecular GmbH	Cat. # 1038100100-STM
Agencourt Ampure XP Beads	Beckman Coulter	Cat. # A63881
Whole Blood Cell Stabilizer Kit	Cytodelics AB	Cat. # mC001-1000mL

TaqMan Vaginal Microbiota Amplification Control	ThermoFisher	Cat. # A32912
TaqMan MGB Probe	ThermoFisher	Cat. # 4316034
Experimental Models: Organisms/Strains		
Mice: C56Bl/6J	The Jackson Laboratory	Stock No: 000664
Mice: 129S1/SvImJ	The Jackson Laboratory	Stock No: 002448
Oligonucleotides		
Forward primer Jarid1 5′-TGAAGCTTTTGGCTTTGAG-3′	This paper	N/A
Reverse primer Jarid1 5′-CCGCTGCCAAATTCTTTGG-3′	This paper	N/A
Software and Algorithms		
R version 4.0.2	R Core Team, 2020	https://www.r-project.org/
RStudio version 1.2.5001	RStudio, Inc.	https://www.rstudio.com/
speciateIT	NA	https://github.com/Ravel-Laboratory/speciateIT
Bioconductor	NA	https://www.bioconductor.org/
kallisto	NA	https://pachterlab.github.io/kallisto/
tximport	NA	https://bioconductor.org/packages/release/bioc/html/tximport.html
edgeR	NA	https://bioconductor.org/packages/release/bioc/html/edgeR.html
limma	NA	https://www.bioconductor.org/packages/release/bioc/html/limma.html
The Astrolabe Cytometry Platform	NA	https://www.astrolabediagnostics.com/
Cytobank	NA	https://www.cytobank.org/
viSNE	NA	https://www.cytobank.org/
FlowSOM	NA	https://www.cytobank.org/
Diet and Glucose Monitor		
Contour Next Blood Glucose Monitoring System	Bayer	Cat. # 0193-9763-01
Low-Fat High-Fiber Diet	LabDiet	Cat. # 5001
High-Fat Low-Fiber Diet	Research Diets	Cat. # D12492

Supplementary Figure 1

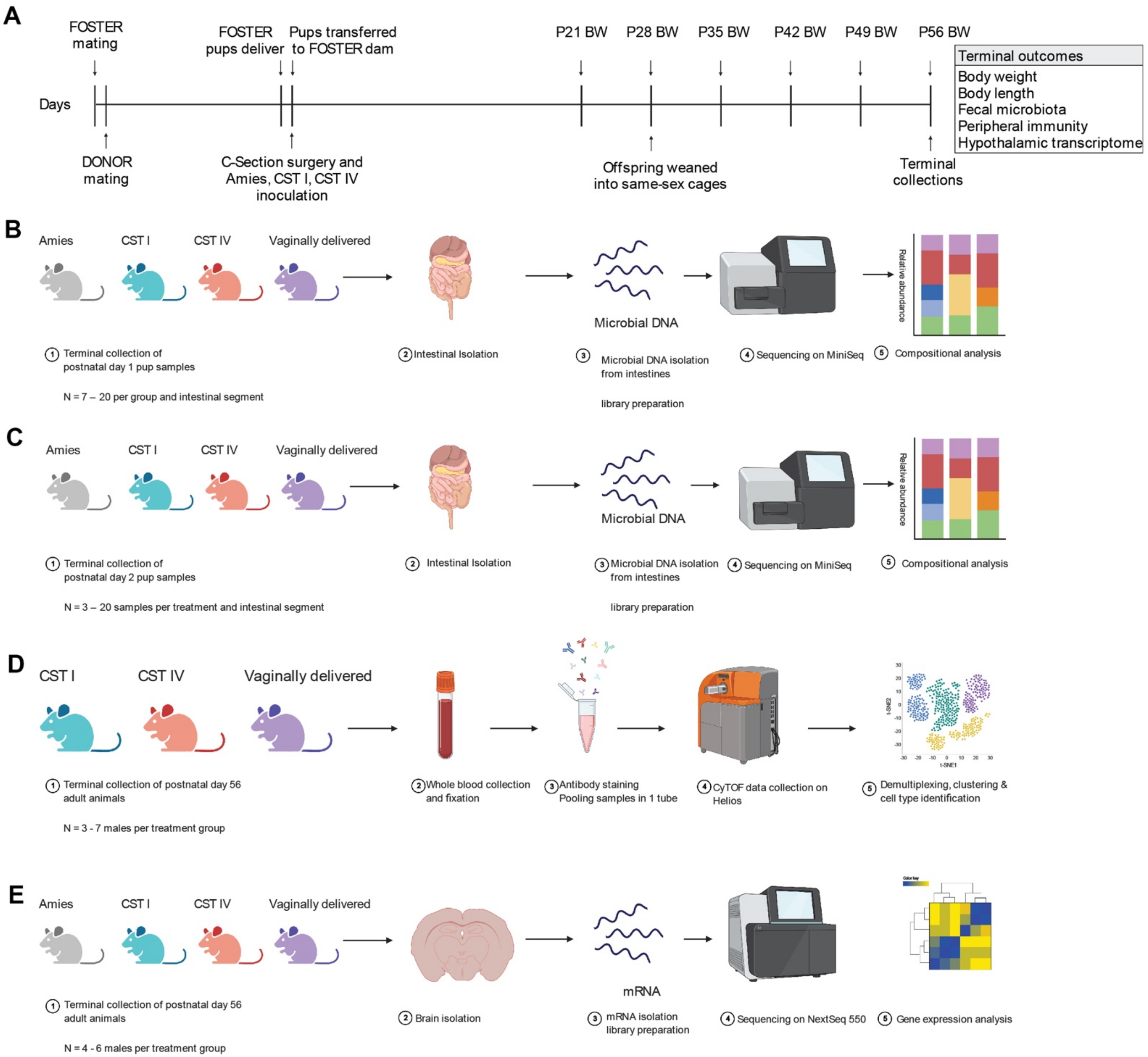

Supplementary Figure 1. Experimental schematics to assess the impact of distinct maternal microbial communities on offspring outcomes.

(**A**) Timeline for all C-section and oral gavage experiments to assess the impact of CST I and CST IV on offspring outcomes, with specific relevance to proof-of-concept experiments depicted in Figure 1 and Supplemental Figure 3-6. Offspring were inoculated with Amies, CST I, or CST IV and transferred to the same foster dam, along with vaginally delivered pups donated from a treatment-naïve donor dam. A subcohort of pups were culled at postnatal day 1 for 16S rRNA gene marker sequencing, and a separate subcohort of pups were culled at postnatal day 2 for microbiome sequencing 16S rRNA gene marker sequencing. Litters contained 8 pups, with 1 male and 1 female from each treatment group represented. Pups were left undisturbed until postnatal day 21, upon which weekly body weight measurements were collected. On postnatal day 28, pups were weaned into same-sex cages. On postnatal day 56, offspring were culled, and terminal body weight and body length was collected. Additionally, fecal pellets, whole blood, and brains were collected for downstream analysis. P, postnatal days. BW, Body Weight. Created with BioRender.com.

(**B**) Schematic of experiments depicted in Figure 1b and Supplemental Figures 2. Samples collected were on postnatal day 1 to measure microbial composition across offspring intestinal segments using 16S rRNA marker gene sequencing or additional verification by qPCR. Twenty-four hours following C-section, inoculation and transfer to foster dam, offspring were culled, and intestinal segments were collected for 16S rRNA gene marker sequencing.

(**C**) Schematic of experiments depicted in Figure 1b and Supplemental Figure 3. Samples were collected on postnatal day 2 to measure microbial composition across offspring intestinal segments using 16S rRNA marker gene sequencing. Forty-eight hours following C-section, inoculation and transfer to foster dam, offspring were culled, and intestinal segments were collected for 16S rRNA gene marker sequencing.

(**D**) Schematic of experiments depicted in Figure 1d-h and Supplemental Figure 6. Samples were collected collected on postnatal day 56 to measure circulating immunity using CyTOF. Terminal whole blood was collected, stabilized, followed by barcoding, multiplexing, and antibody staining, data collection on the Helios, and downstream analysis, as detailed in *Methods*.

(**D**) Schematic of experiments depicted in Figure 1i-j. Samples were collected on postnatal day 56 to measure gene expression patterns in the paraventricular nucleus of the hypothalamus. Offspring brains were rapidly excised and frozen on dry ice. PVN micropunches were collected, mRNA was isolated, cDNA libraries were prepared, sequenced, and downstream gene expression analysis was conducted.

Supplementary Figure 2

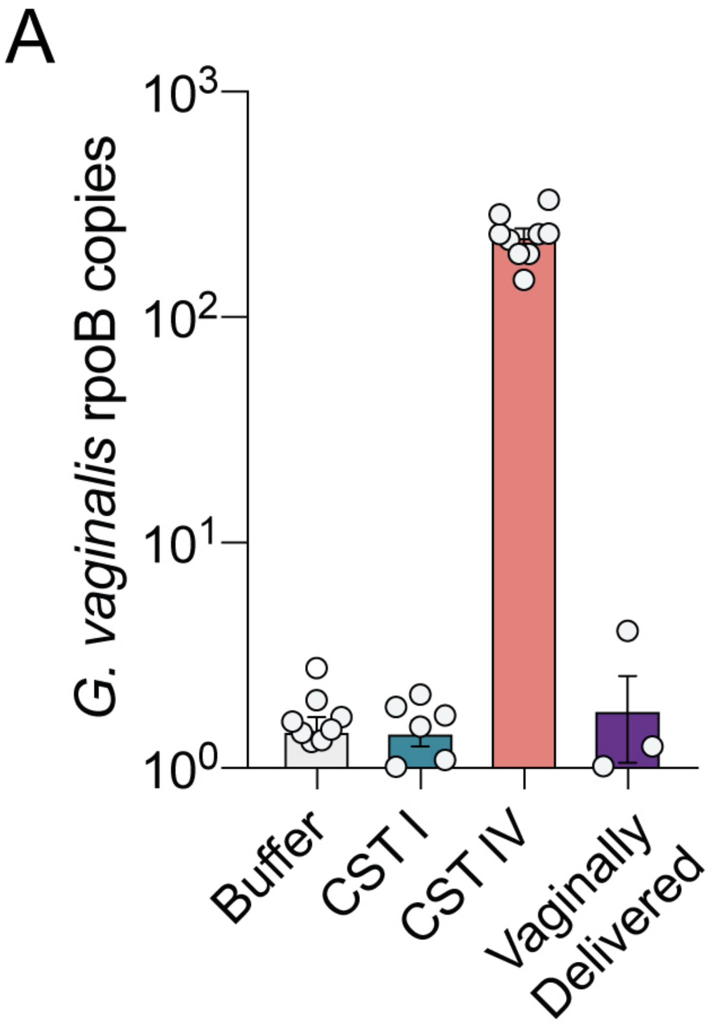

Supplementary Figure 2. Quantitative PCR-based validation of human vaginal microbiota in the intestine of C-section delivered mice exposed to Amies, CST I or CST IV or vaginally delivered pups.

(**A**) C-section mice inoculated with CST IV show increased rpoB copy number compared with Amies, CST I and vaginally delivered offspring. Copy number of *G. vaginalis* was quantified using TaqMan assay for *rpoB*, and the TaqMan vaginal microbiota amplification control was used to evaluate amplification efficiency. N = 8 Amies, 6 CST I, 9 CST IV, 3 vaginally delivered. Data represented as mean ± SEM with individual data points overlaid.

Supplementary Figure 3

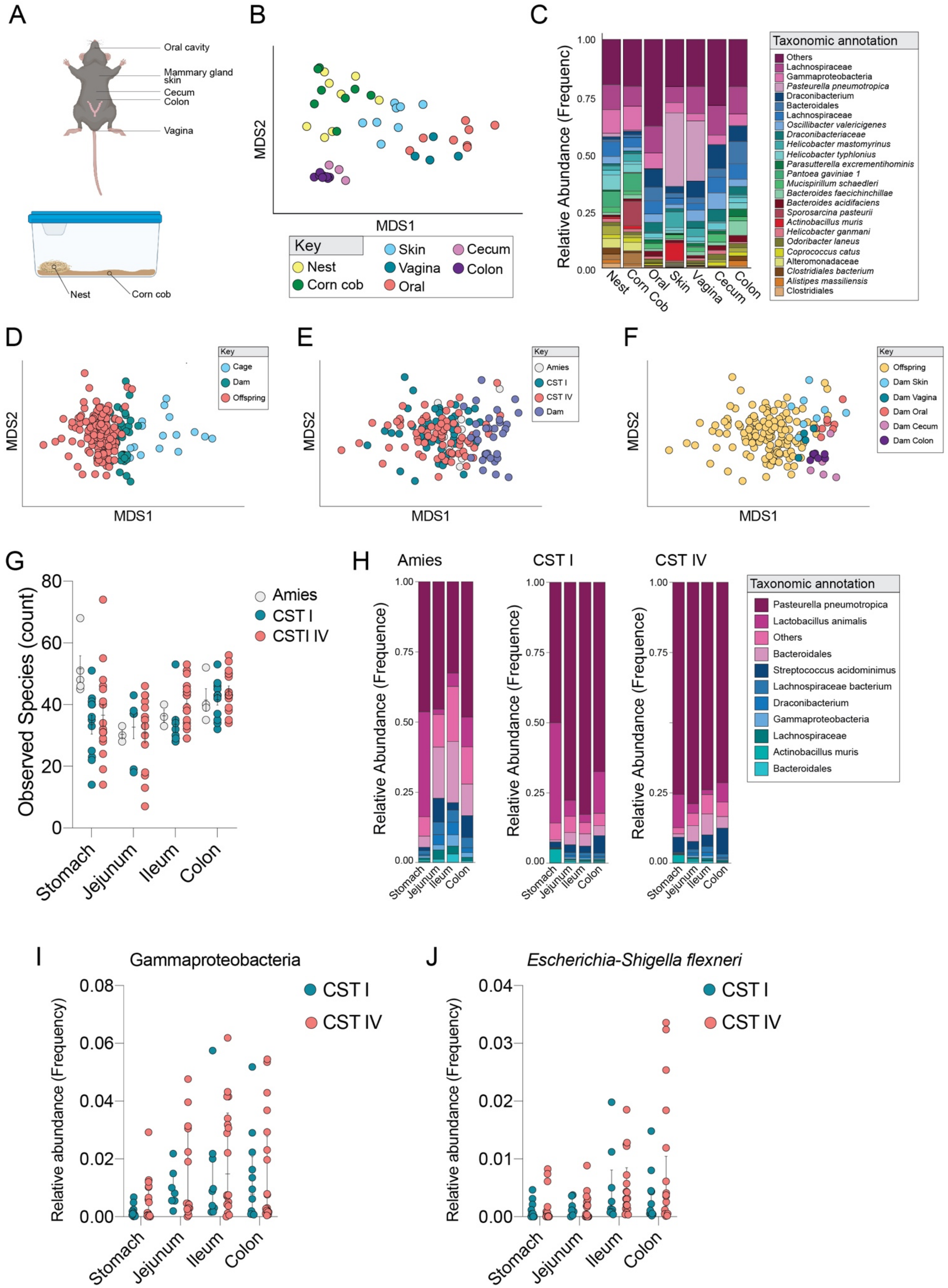

Supplementary Figure 3. Characterizing the environmental and maternal microbial reservoirs that contribute to microbial assembly in C-section offspring.

(**A**) Schematic of the samples collected to determine the microbial reservoirs involved in vertical transmission. The local cage environment was assessed by sampling nestlet material on which pups had direct physical contact, and corn cob was sampled surrounded the nestlet. Maternal samples were collected as depicted in the schematic. Created with BioRender.com.

(**B**) Non-metric multidimensional scaling (NMDS) analysis of environmental and maternal samples showing that clustering was influenced by maternal body site.

(**C**) Mean relative abundance of the top 20 taxa across maternal body sites and the local cage environment, demonstrating a bloom of *Pasteurella* recovered from the mammary gland associated skin and vagina of the foster dam.

(**D**) NMDS analysis of offspring, foster dam, and cage samples showing distinct clustering of offspring and cage samples, with foster dam as an intermediary. N = 3 – 15 samples per treatment and intestinal segment; 15 cage samples; 32 foster dam samples.

(**E**) NMDS analysis showing no distinct clustering between gut samples from Amies, CST I and CST IV inoculated C-section pups. N = 128 C-section pup samples; 32 foster dam samples.

(**F**) NMDS analysis showing closest clustering between C-section pup gut samples and the foster dams compared with samples from the vagina, cecum, colon, and oral cavity of the foster dam. N = 128 C-section pup samples; 32 foster dam samples.

(**G**) Barplot demonstrating intestinal region-specific differences in alpha diversity in Amies, CST I, and CST IV C-section pups. N = Stomach: 5 Amies, 11 CST I, 15 CST IV; Jejunum: 3 Amies, 5 CST I, 13 CST IV; Ileum: 3 Amies, 6 CST I, 14 CST IV; Colon: 4 Amies, 10 CST I, 13 CST IV samples that passed quality filtering. Data represented as mean ± SEM with individual data points overlaid.

(**H**) Mean relative abundance of the top 20 taxa across intestinal segments between Amies, CST I, CST IV C-section pups demonstrating that *Pasteurella* as a dominant taxon in the neonate gut. N = 3 – 15 samples per treatment and intestinal segment.

(**I**) Linear discriminant analysis - Effect Size (LEfSe) analysis demonstrating increased abundance of Gammproteobacteria in CST IV inoculated C-section offspring in an intestinal region-specific manner (FDR = 0.007, LDAscore = 4.86). N = Stomach: 11 CST I, 15 CST IV; Jejunum: 5 CST I, 13 CST IV; Ileum: 6 CST I, 14 CST IV; Colon: 10 CST I, 13 CST IV samples that passed quality filtering. Data represented as median ± IQR with individual data points overlaid.

(**J**) Linear discriminant analysis - Effect Size (LEfSe) analysis demonstrating increased abundance of Escherichia-Shigella in CST IV inoculated C-section offspring in an intestinal region-specific manner (FDR = 0.0003, LDAscore = 4.34). N = Stomach: 11 CST I, 15 CST IV; Jejunum: 5 CST I, 13 CST IV; Ileum: 6 CST I, 14 CST IV; Colon: 10 CST I, 13 CST IV samples that passed quality filtering. Data represented as median ± IQR with individual data points overlaid.

Supplementary Figure 4

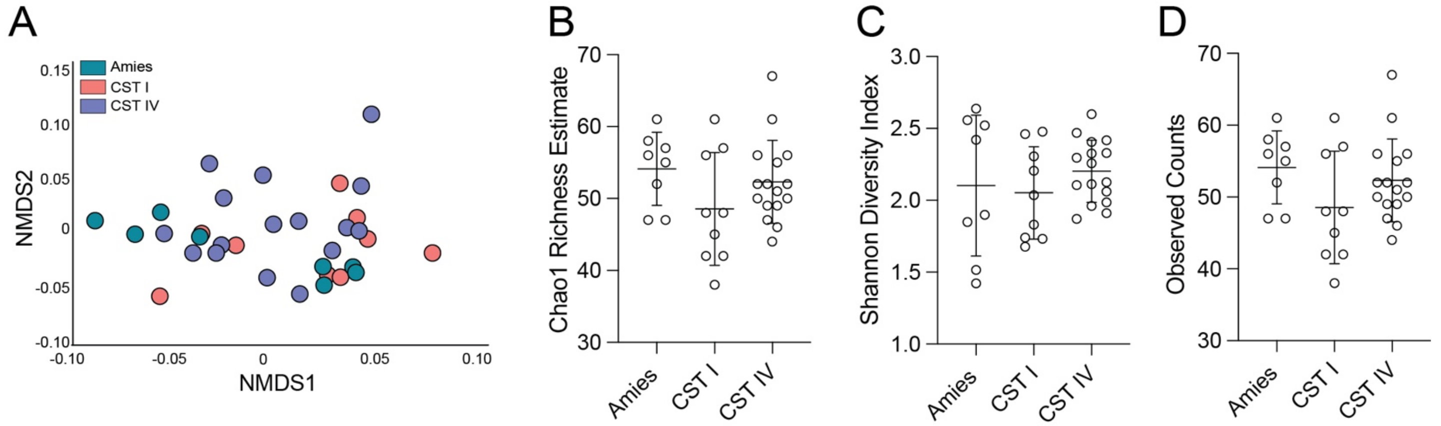

Supplementary Figure 4. Fecal microbiota composition in adulthood is similar between Amies, CST I and CST IV inoculated adult males.

(**A**) NMDS analysis showing similarity in community structure in Amies, CST I and CST IV inoculates males in adulthood (PERMANOVA, F-value = 1.7458, R^2 =0.1043, p = 0.134). N = 8 Amies males, 9 CST I males, 16 CST IV males.

(**B**) Barplot demonstrating similarities in the alpha diversity measure Chao1 Richness Estimate between Amies, CST I, and CST IV C-section adult males (One-way ANOVA, F-value = 1.82, p = 0.17). N = 8 Amies males, 9 CST I males, 16 CST IV males. Data represented as mean ± SD with individual data points overlaid.

(**C**) Barplot demonstrating similarities in the alpha diversity measure Shannon Diversity Index between Amies, CST I, and CST IV C-section adult males (One-way ANOVA, F-value = 0.66, p = 0.52). N = 8 Amies males, 9 CST I males, 16 CST IV males. Data represented as mean ± SD with individual data points overlaid.

(**D**) Barplot demonstrating similarities in the alpha diversity measure Observed Counts between Amies, CST I, and CST IV C-section adult males (One-way ANOVA, F-value = 1.82, p = 0.18). N = 8 Amies males, 9 CST I males, 16 CST IV males. Data represented as mean ± SD with individual data points overlaid.

Supplementary Figure 5

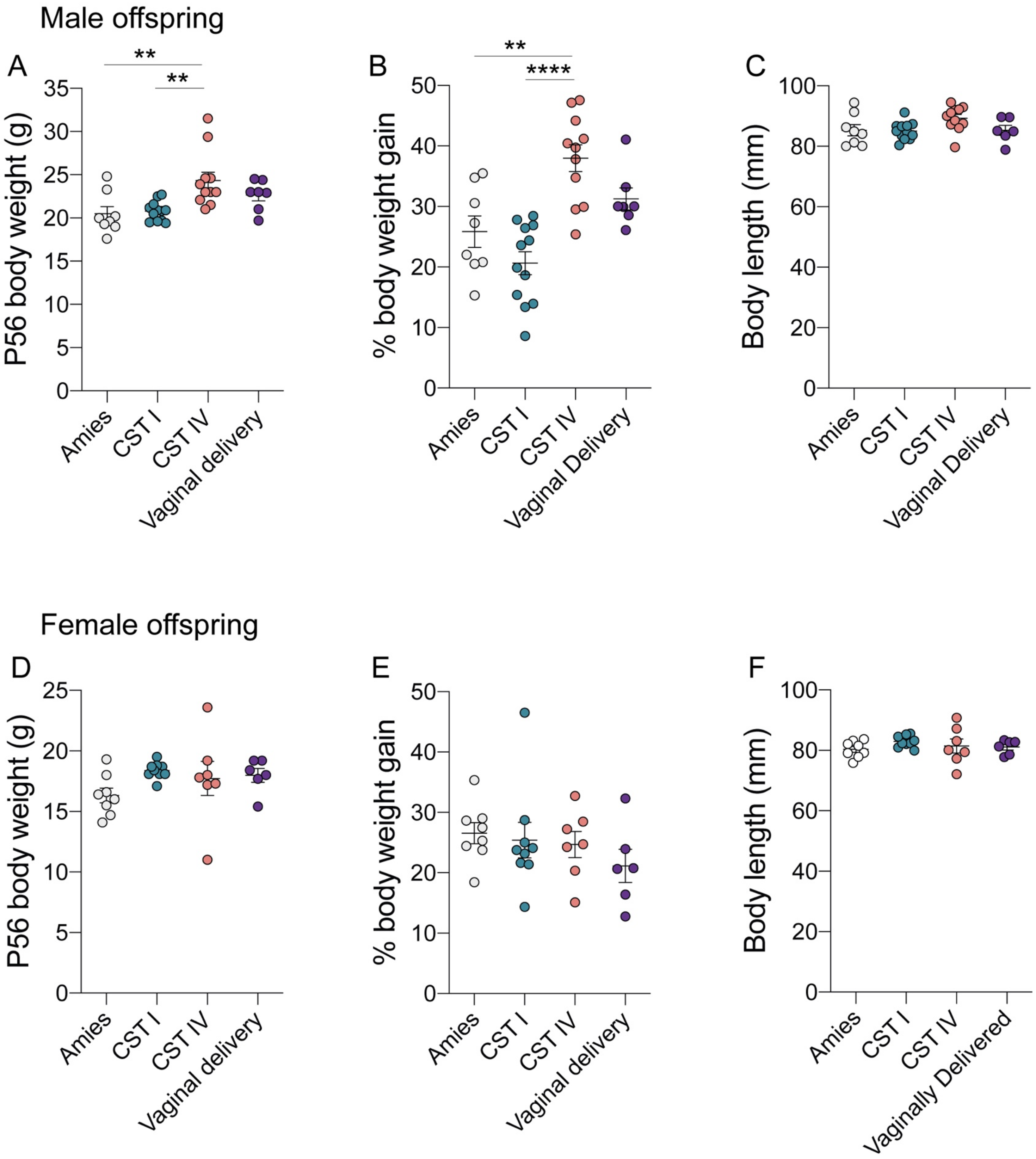

Supplementary Figure 5. Exposure to human vaginal microbiota associates with body weight measurements in a sex-specific manner.

(**A**) CST IV males weighed more than CST I and Amies males (one-way ANOVA, main effect of treatment, $F_{3, 34} = 6.280$, P=0.0017); Tukey's post-hoc, CST I vs. CST IV, $P = 0.0039$; Amies vs. CST IV, $P = 0.0050$). N = 7 Amies, 12 CST I, 11 CST IV, 7 vaginally delivered males. Data represented as mean ± SEM with individual data points overlaid. ** $P < 0.01$.

(**B**) CST IV males showed increased percent body weight gain from P28 to P56 compared with CST I and Amies males $F_{3, 34} = 13.58$, $P < 0.0001$; Tukey's post-hoc, CST I vs. CST IV, $P < 0.0001$; Amies vs. CST IV, $P = 0.0024$). N = 7 Amies, 12 CST I, 11 CST IV, 7 vaginally delivered males. Data represented as mean ± SEM with individual data points overlaid. ** $P < 0.01$, **** $P < 0.0001$.

(**C**) Body weight differences were not due to differences in body length between Amies, CST I, CST IV, and vaginally delivered males at P56. N = 7 Amies, 12 CST I, 11 CST IV, 7 vaginally delivered males. Data represented as mean ± SEM with individual data points overlaid.

(**D to F**) No differences in P56 body weight, percent body weight gain, or body length were observed between Amies, CST I, CST IV, and vaginally delivered female offspring. N = 8 Amies, 9 CST I, 7 CST IV, 6 vaginally delivered females. Data represented as mean ± SEM with individual data points overlaid.

Supplementary Figure 6.

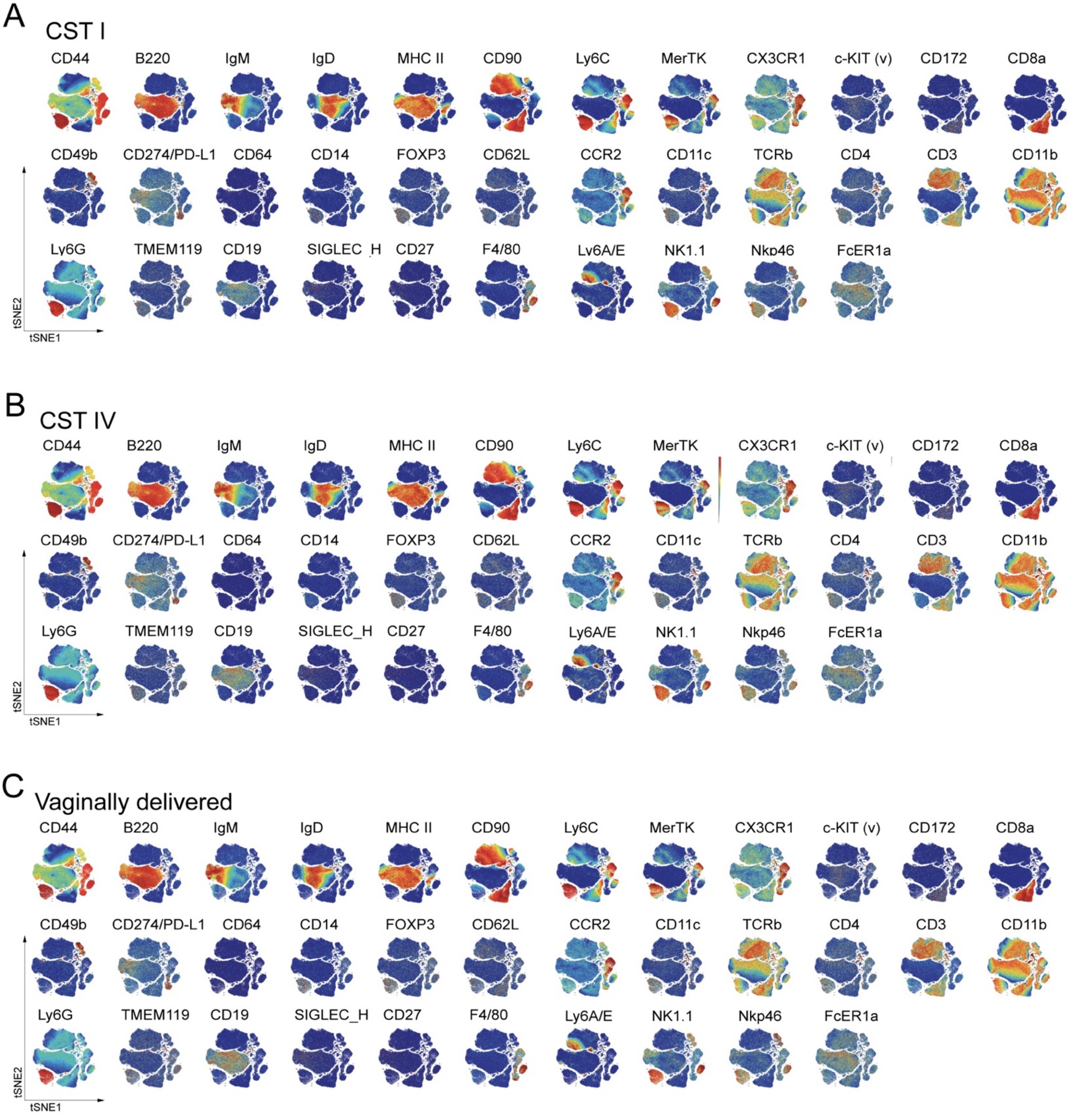

Supplementary Figure 6. High-dimensional analysis of mass cytometry data using viSNE comparing the lasting effects of mode of delivery and birth-associated exposure to human vaginal microbiota on the circulating immune compartment.

(A to C) High-dimensional analysis of mass cytometry data using viSNE comparing the lasting effects of mode of delivery and colonization by human vaginal microbiota on the circulating immune compartment of cesarean delivered males inoculated with (a) CST I or (b) CST IV and (c) vaginally delivered males at postnatal day 56. viSNE maps were generated using equal sampling across treatment groups and the markers shown were used to generate the maps (n= 390,000 total events sampled, representative average from N = 3 -7 males per treatment).

Supplementary Figure 7

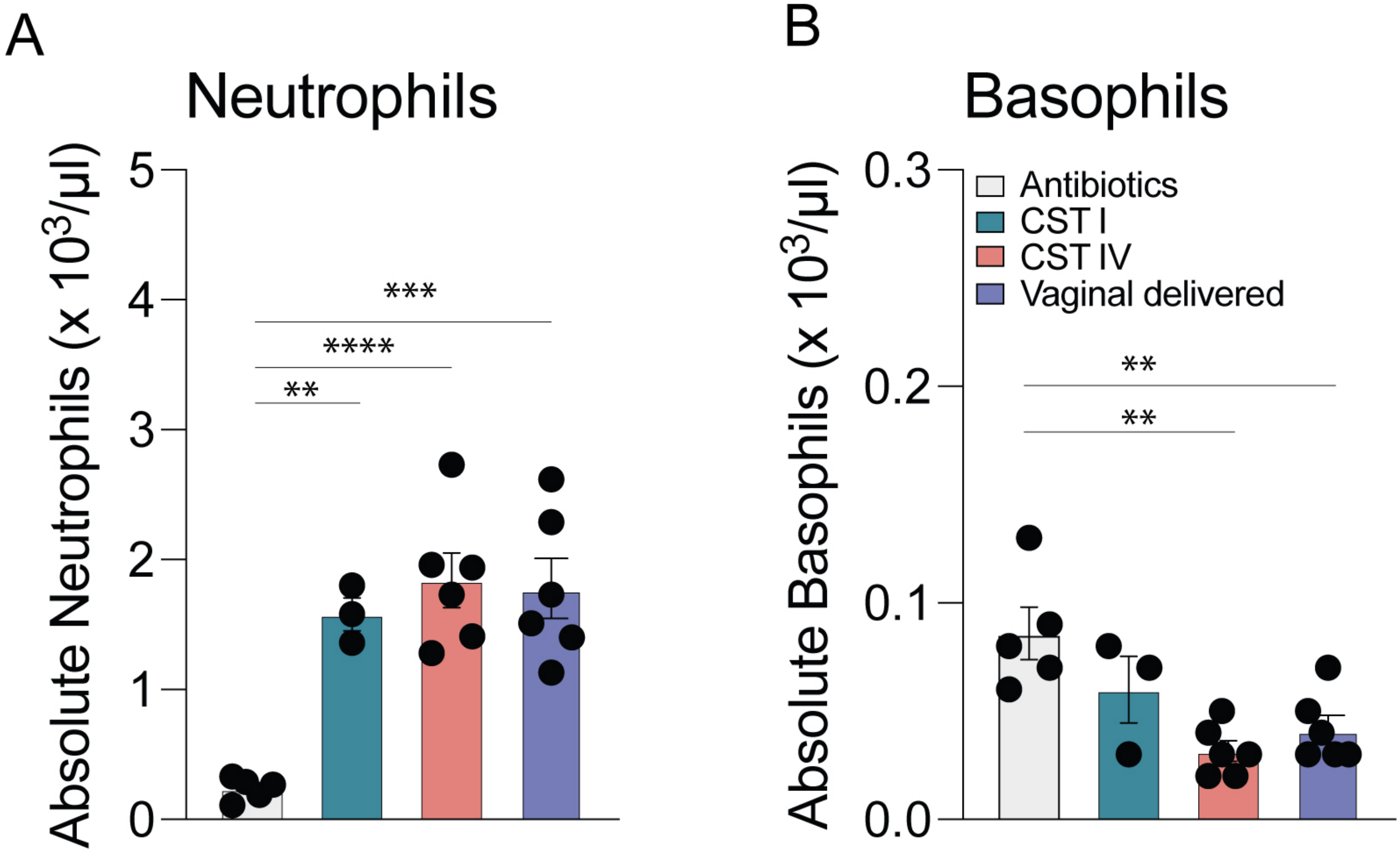

Supplementary Figure 7. Birth-associated exposure to microbiota influences number of circulating neutrophils and basophils in 24-hour old males.

(**A**) To determine whether early life colonization is necessary for expansion of neutrophil counts in circulation, we exposed pregnant dams to a clinically relevant combination of ampicillin, gentamicin and vancomycin (all 1 mg ml^{-1}) starting at gestational day 15 to postnatal day 1. On postnatal day (P) 1, corresponding to 24 hr post-inoculation, absolute white blood cell counts were quantified on an Element HT5 veterinary hematology analyzer. Neutrophil count is significantly decreased in P1 antibiotic-exposed males compared with CST I, CST IV and vaginally delivered males (one-way ANOVA, main effect of treatment, $F_{3, 16} = 15.46$, $P < 0.0001$; Tukey's post-hoc, Antibiotics vs. CST I, $P = 0.0034$; Antibiotics vs. CST IV $P < 0.0001$; P2 Antibiotics vs. VD $P = 0.0001$). N = 5 Antibiotics males, 3 CST I males, 6 CST IV males, 6 vaginally delivered males. Data are represented as mean ± SEM. ** $P < 0.01$, *** $P < 0.001$, **** $P < 0.0001$.

(**B**) Basophil count is significantly increased in P1 antibiotic-exposed males compared with CST IV and vaginally delivered males (one-way ANOVA, main effect of treatment, F (3, 16) = 7.710, P=0.002; Tukey's post-hoc, Antibiotics vs. CST IV $P = 0.0018$; P2 Antibiotics vs. VD $P = 0.0096$). N = 5 Antibiotics males, 3 CST I males, 6 CST IV males, 6 vaginally delivered males. Data are represented as mean ± SEM. ** $P < 0.01$.

Supplementary Figure 8

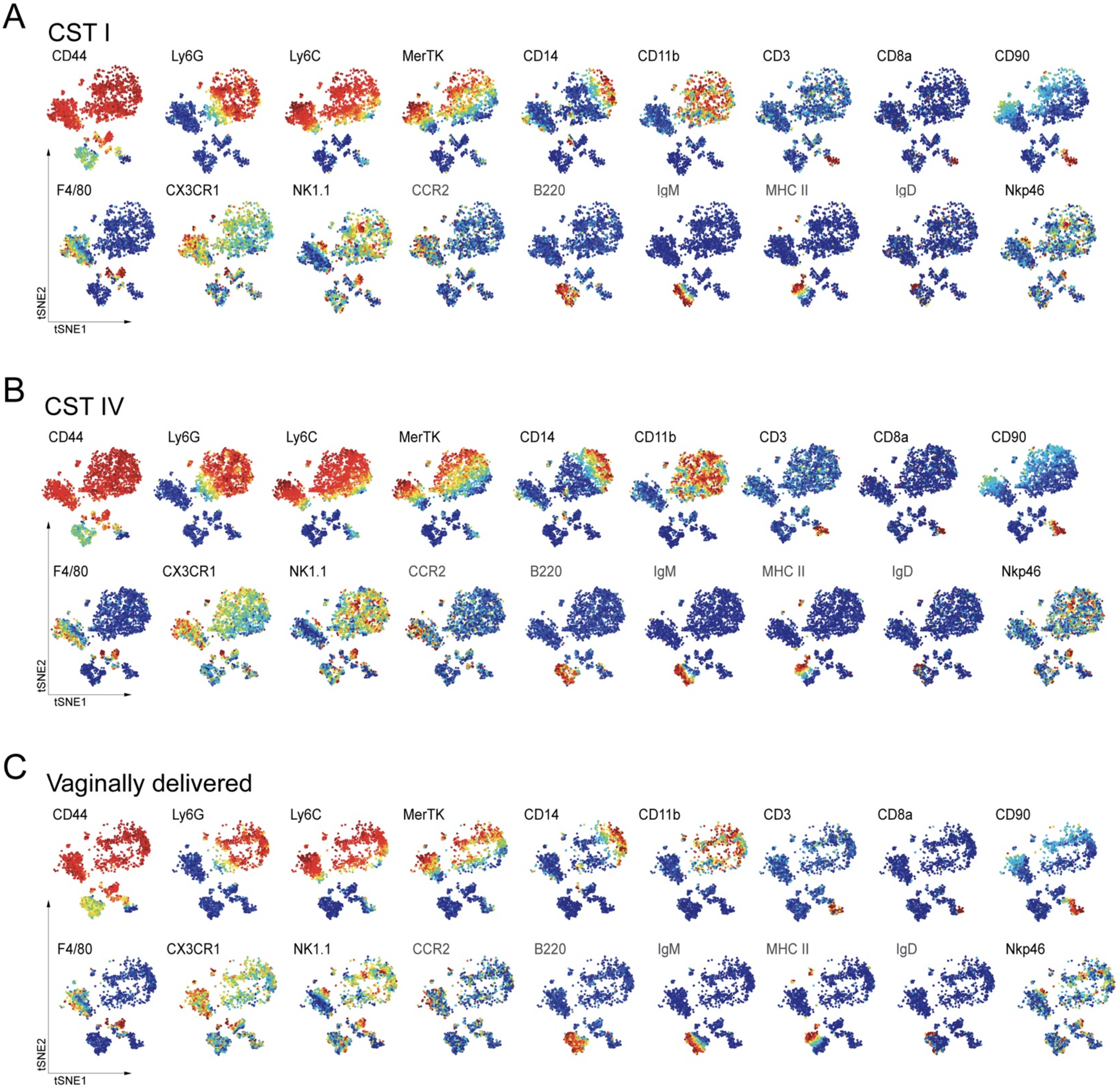

Supplementary Figure 8. High-dimensional analysis of circulating immune compartment following birth-associated exposure to microbiota.

(**A to C**) High-dimensional analysis of mass cytometry data using viSNE comparing the circulating immune compartment of cesarean delivered males inoculated with human vaginal (a) CST I or (b) CST IV and (c) vaginally delivered males at postnatal day 1, corresponding to 24 hrs post-inoculation. viSNE maps were generated using equal sampling and the markers shown were used to generate the maps (n = 10,000 total events sampled, representative average from N = 3 males per treatment).

Supplementary Figure 9

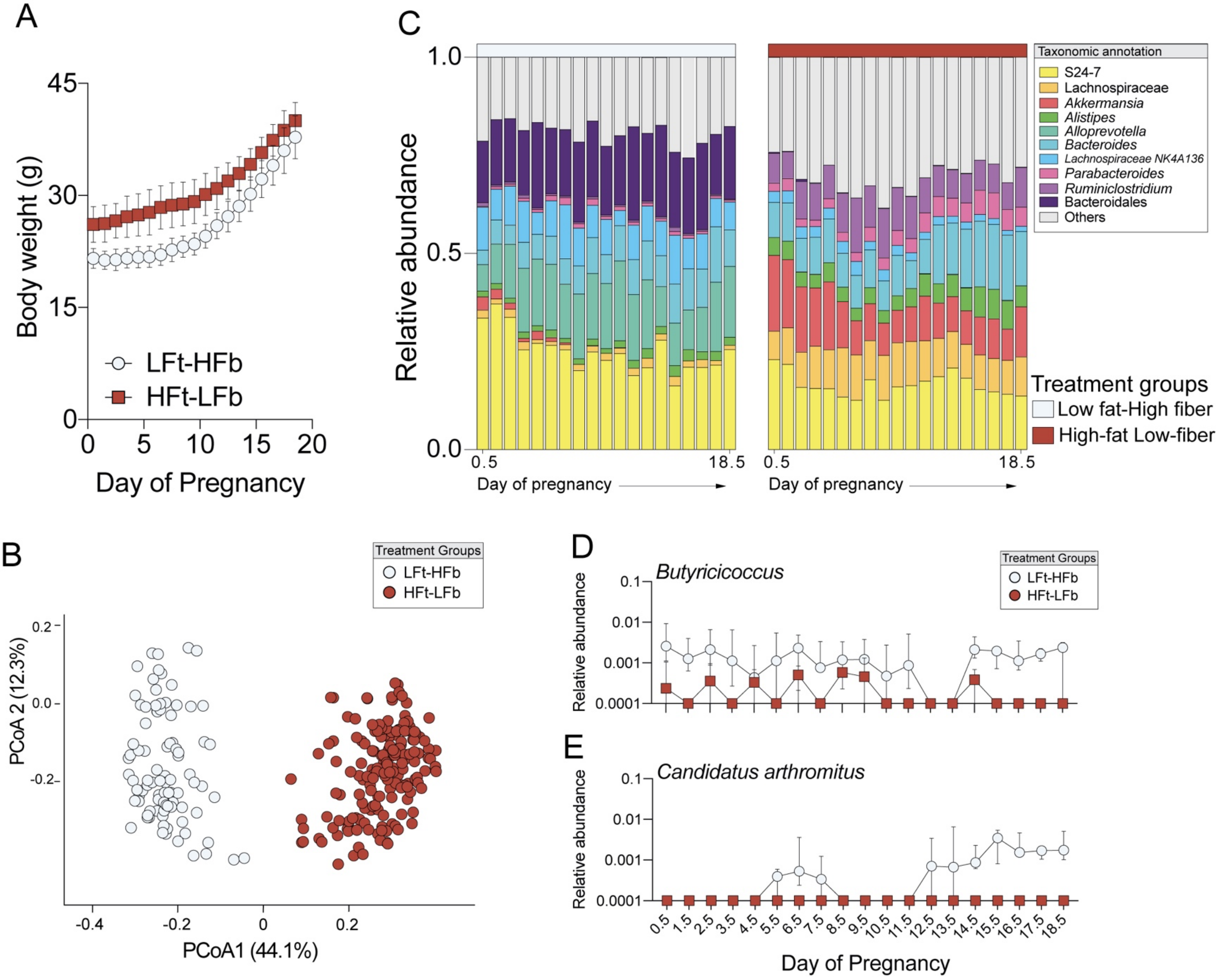

Supplementary Figure 9. Maternal consumption of a high fat-low fiber diet results in persistent disruption to body weight and gut microbiota during pregnancy.

(**A**) Excessive maternal weight gain during pregnancy in dams consuming a high-fat low-fiber diet (two-way ANOVA, main effect of time, $F_{18,324} = 474.24$, $P < 0.0001$; main effect of diet, $F_{1,18} = 23.51$, $P < 0.0001$; time*diet interaction, $F_{18,324} = 6.73$, $P < 0.0001$). No effect of *G. vaginalis* 11E4 on body weight was observed, thus samples are shown collapsed by diet. N = 8 LFt-HFb dams, 12 HFt-LFb dams per timepoint. Data represented as mean ± SEM with individual datapoints overlaid. Data is representative of two independent experiments

(**B**) Principal coordinates analysis demonstrating distinct clustering between dams consuming a high-fat low fiber and low-fat high-fiber diet across pregnancy. No effect of *G. vaginalis* 11E4 on gut microbiota structure was observed, thus samples are shown collapsed by diet. N = 12 – 20 females per treatment and timepoint, total of 259 samples.

(c) Mean relative abundance of top ten lasting disruption to the fecal microbiota during pregnancy in females consuming a high-fat low-fat diet. No effect of *G. vaginalis* 11E4 on gut microbiota composition was observed, thus samples are shown collapsed by diet. N = 12 – 20 females per treatment and timepoint, total of 259 samples.

(**D**) LEfSe analysis demonstrating decreased relative abundance of the butyrate-producing taxa *Butyricoccus* in females consuming a high-fat low-fat diet (FDR = , LDAscore =). No effect of *G. vaginalis* 11E4 on *Butyricoccus* relative abundance was observed, thus samples are shown collapsed by diet. N = 8 LFt-HFb dams, 12 HFt-LFb dams per timepoint., total of 259 samples that passed quality filtering. Data represented as median ± IQR with individual data points overlaid.

(**E**) LEfSe analysis demonstrating decreased abundance of the immunomodulatory *Candidatus Arthromitus* in females consuming a high-fat low-fat diet during late pregnancy (FDR = , LDAscore =). No effect of *G. vaginalis* 11E4 on *Candidatus Arthromitus* relative abundance was observed, thus samples are shown collapsed by diet. N = 8 LFt-HFb dams, 12 HFt-LFb dams per timepoint., total of 259 samples that passed quality filtering. Data represented as median ± IQR with individual data points overlaid.

Supplementary Figure 10

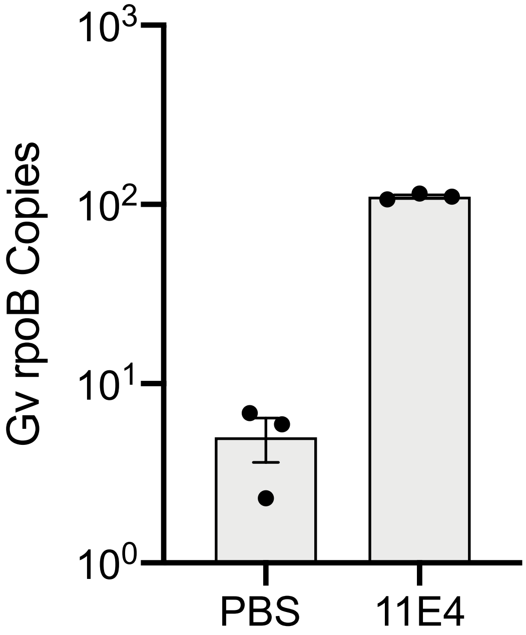

Supplementary Figure 10. Confirmation of *G. vaginalis* isolate 11E4 in vaginal fluid 48hrs post-colonization.

Presence of Gv was significantly increased in the vaginal fluid from Gv inoculated dams relative to PBS inoculated dams (Unpaired two-sided t-Test, $t_4 = 36.45$, $P < 0.0001$). N = 3 PBS inoculated dams, 3 Gv 11E4 inoculated dams. Data represented as mean ± SEM with individual data points overlaid.

Supplementary Figure 11

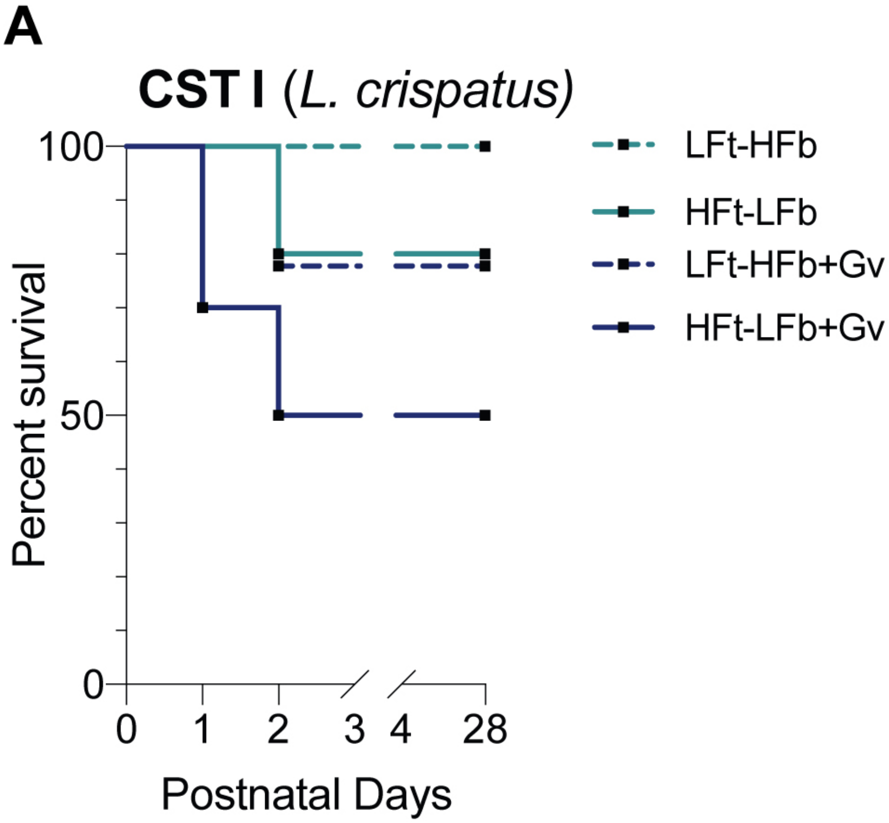

Supplementary Figure 11. Combined effects of exposure to compounding maternal insults and colonization by human vaginal CST I on offspring survival.

(**A**) Survival of offspring from dams that experience a single or multiple compounding adversities. All pups were C-section delivered and gavaged with human CST I inoculant. LFt-HFb = low-fat high-fiber; HFt-LFb = high-fat low-fiber; Gv = G. vaginalis 11E4. Kaplan-Meier survival analysis. N=15 offspring per treatment condition.

Supplementary Figure 12

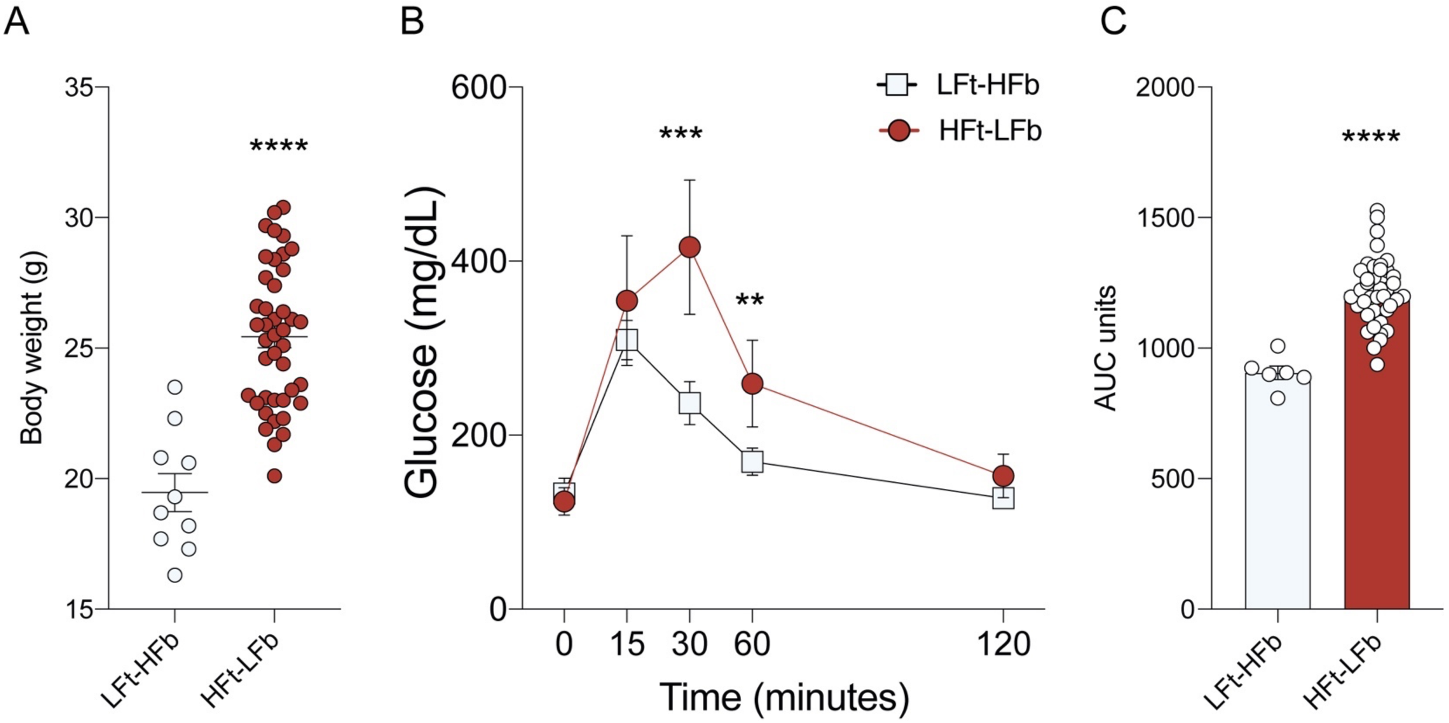

Supplementary Figure 12. Replication cohort confirms excessive maternal weight gain and glucose intolerance in females consuming a high fat-low fiber diet for six weeks.

(**A**) Consumption of a high-fat low-fiber diet for six weeks increases body weight in females consuming a high-fat low-fiber diet compared with females consuming a low-fat high-fiber diet (two-tailed t-Test, $t_{50} = 6.408$, $P < 0.0001$). N = 10 LFt-HFb females, 42 HFt-LFb females. Data represented as mean ± SD. **** $P < 0.0001$.

(**B**) Plasma levels of glucose levels during a glucose tolerance test in females consuming either a high-fat low-fiber or low-fat high-fiber diet. Females consuming a high-fat low-fiber diet showed significant delay in glucose clearance (two-way ANOVA, main effect of time, $F_{4,\,180} = 84.09$, $P < 0.0001$; main effect of diet, $F_{1,\,45} = 29.65$, $P < 0.0001$; time*diet interaction, $F_{4,\,180} = 11.56$, $P < 0.0001$). N = 6 LFt-HFb females, 36 HFt-LFb females. Data represented as mean ± SD. ** $P < 0.01$; *** $P < 0.001$.

(**C**) AUC of total plasma glucose levels showing increase glucose levels in females consuming a high-fat low-fiber diet (two-tailed T test, $t_{44} = 5.821$, $P < 0.0001$). N = 6 LFt-HFb females, 36 HFt-LFb females. Data represented as mean ± SD with individual datapoints overlaid. **** $P < 0.0001$.

Supplementary Figure 13

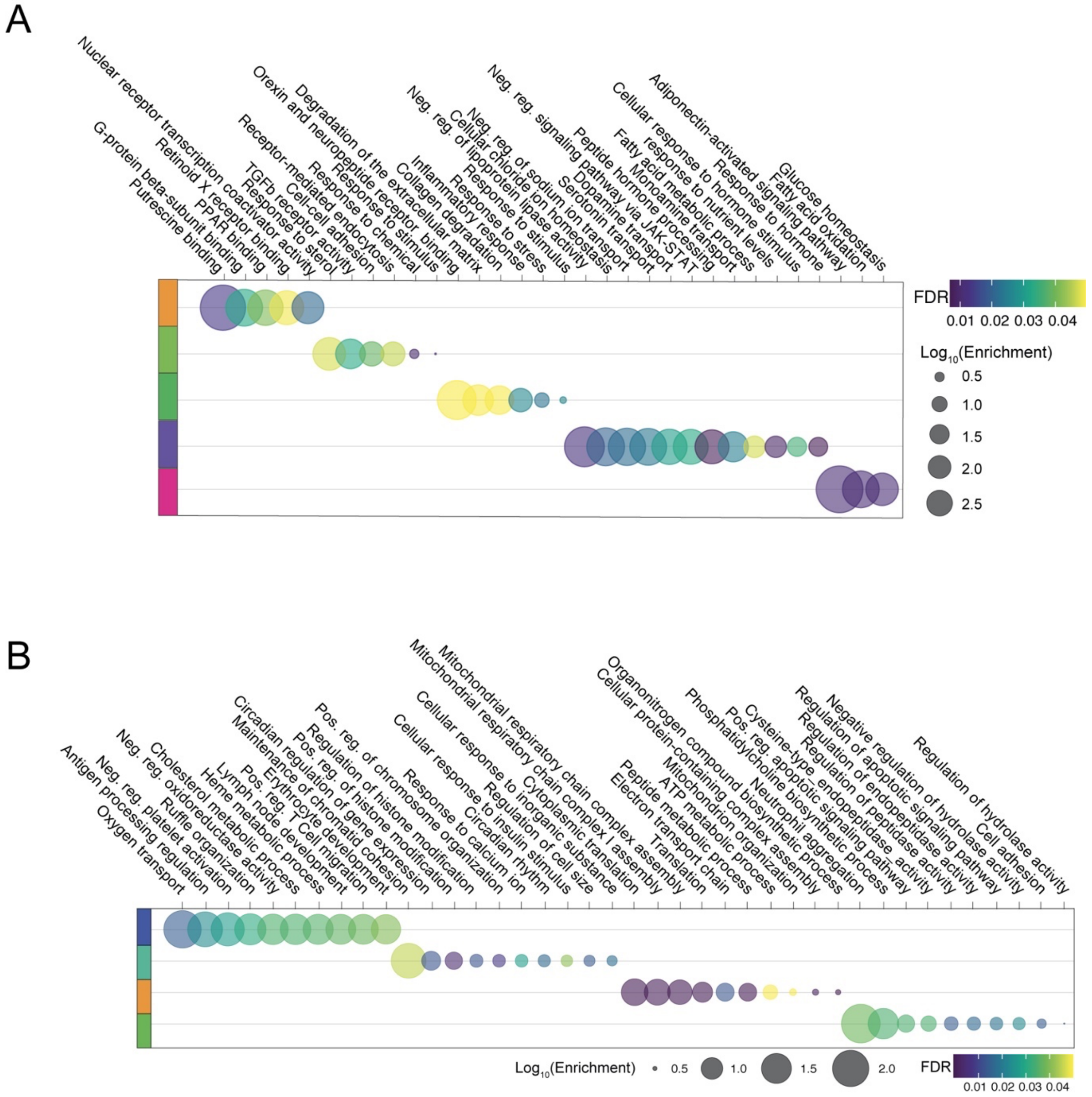

Supplementary Figure 13. Cluster-based functional enrichment analysis showing significant effect of HFt-LFb and *G. vaginalis* and their combination on metabolic and immune pathways in the E18.5 placenta.

(**A**) Cluster-based functional enrichment analysis of differentially expressed genes in the placenta of embryonic day 18.5 males, showing significant disruption in pathways involved in nutrient transport and fatty acid metabolism in the placenta of males exposed to a maternal high-fat low fiber diet and *G. vaginalis* vaginal colonization (FDR < 0.05). Bubble plot size denotes enrichment. Clusters identified in differential gene expression analysis were used for this pathway enrichment. N = 3 males per treatment.

(**B**) Cluster-based functional enrichment analysis of differentially expressed genes in the ileum of embryonic day 18.5 males, showing significant disruption in pathways involved in tissue development, chromatin modification, immunity, and mitochondrial function in the ileum of males exposed to a maternal high-fat low fiber diet and G. vaginalis vaginal colonization (FDR < 0.05). Bubble plot size denotes enrichment. Clusters identified in differential gene expression analysis were used for this pathway enrichment. N = 3 males per treatment.

Supplementary Figure 14

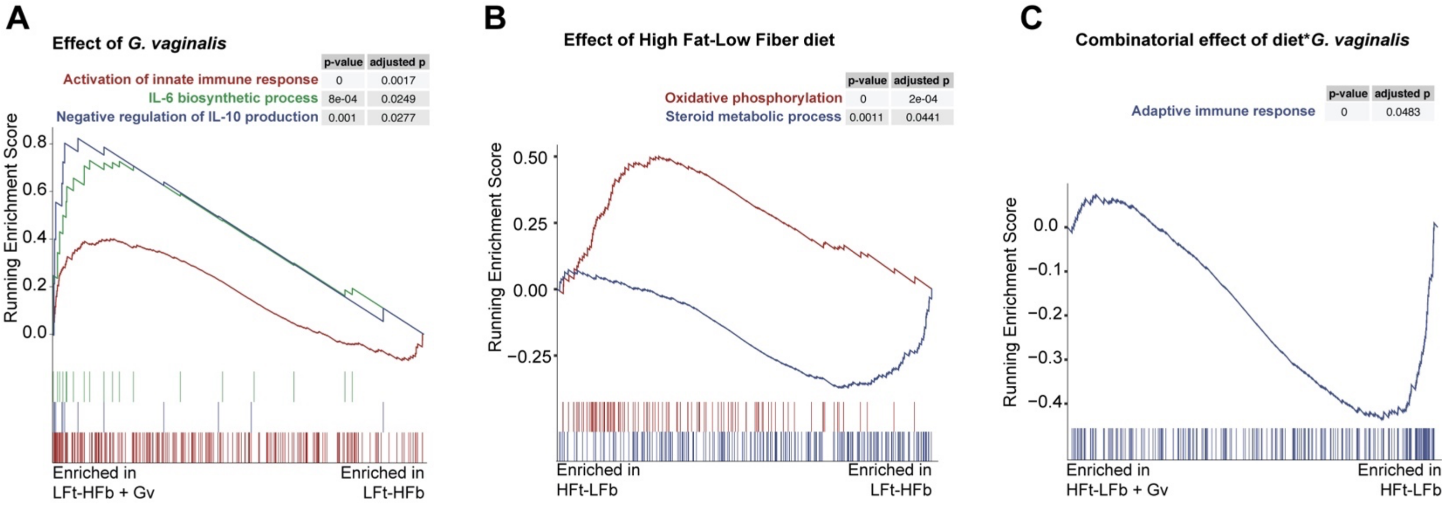

Supplementary Figure 14. Gene set enrichment analysis (GSEA) showing significant effect of HFt-LFb and *G. vaginalis* and their combination on metabolic and immune pathways in the E18.5 placenta.

(**A**) Analysis comparing the effects of *G. vaginalis* in LFt-HFb placentas shows significant enrichment of gene sets involved in activation of innate immune response, IL-6 biosynthesis, and negative regulation of IL-10 production relative to LFt-HFb placentas (N = 3 males per group, GSEA cut off FDR ≤ 0.05, NES > |1.5|).

(**B**) Analysis comparing the effects of diet shows significant dysregulation of gene sets involved in oxidative phosphorylation and steroid metabolic processes in HFt-LFb male placentas relative to LFt-HFb male placentas (N = 3 males per group, GSEA cut off FDR ≤ 0.05, NES > |1.5|).

(**C**) Analysis comparing the interaction between diet and *G. vaginalis* shows significant dysregulation of gene sets involved in the adaptive immune response in HFt-LFb + Gv male placentas relative to LFt-HFb male placentas (N = 3 males per group, GSEA cut off FDR ≤ 0.05, NES > |1.5|).

Supplementary Figure 15

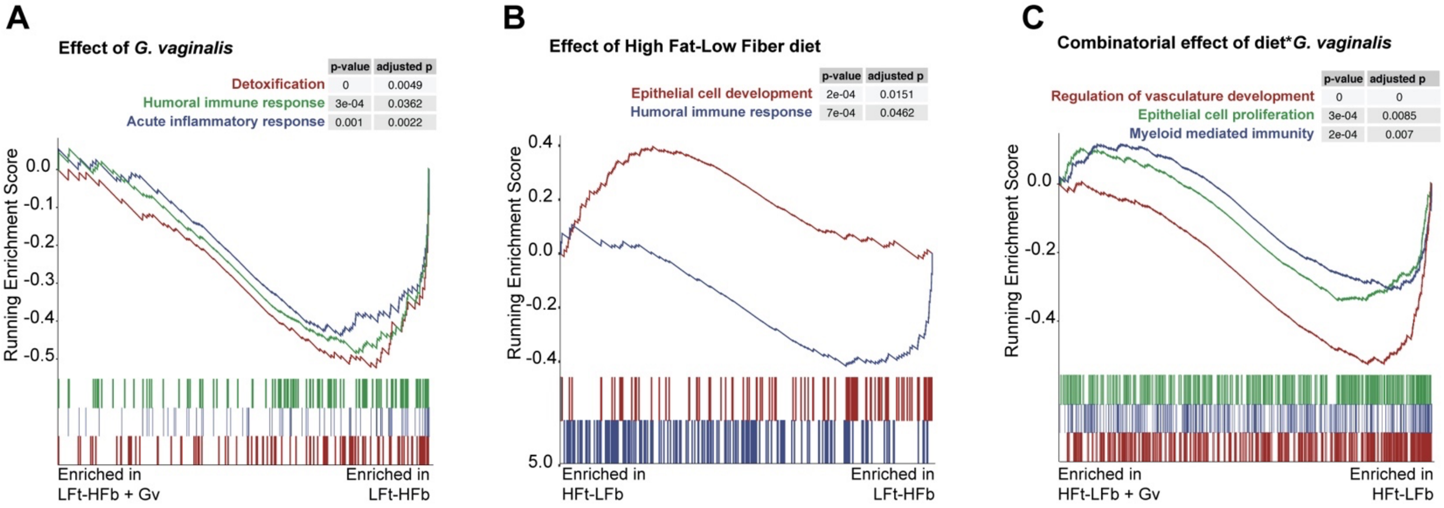

Supplementary Figure 15. Gene set enrichment analysis (GSEA) showing significant effect of HFt-LFb and *G. vaginalis* and their combination on pathways involved in immune, epithelial and vasculature maturation of the E18.5 ileum.

(**A**) Analysis comparing the effects of *G. vaginalis* in LFt-HFb ileum shows disruption in the gene sets involved in metabolic detoxification, the humoral immune response and the acute inflammatory response relative to the ileum of LFt-HFb pups (N = 3 males per group, GSEA cut off FDR $\leq$ 0.05, NES > |1.5|).

(**B**) Analysis comparing the effects of diet shows significant dysregulation of gene sets involved in humoral immune response and epithelial cell development in HFt-LFb male ileum samples relative to LFt-HFb male ileum samples (N = 3 males per group, GSEA cut off FDR $\leq$ 0.05, NES > |1.5|).

(**C**) Analysis comparing the interaction between diet and *G. vaginalis* shows significant dysregulation of gene sets involved in the regulation of vasculature development, epithelial cell proliferation and myeloid mediated immunity in HFt-LFb + Gv male ileum samples relative to LFt-HFb male ileum samples (N = 3 males per group, GSEA cut off FDR $\leq$ 0.05, NES > |1.5|).
