# Supplementary material for: The composition of human vaginal microbiota transferred at birth affects offspring health in a mouse model
Source: Nat Commun. 2021 Nov 1;12:6289. doi: 10.1038/s41467-021-26634-9 (PMC8560944; doi:10.1038/s41467-021-26634-9)
Supplement: Supplementary file 2 — Reporting Summary [file 41467_2021_26634_MOESM2_ESM.pdf]

nature research

Corresponding author(s): Tracy L. Bale, PhD

Last updated by author(s): Oct 6, 2021

Reporting Summary

Nature Research wishes to improve the reproducibility of the work that we publish. This form provides structure for consistency and transparency in reporting. For further information on Nature Research policies, see our Editorial Policies and the Editorial Policy Checklist.

Statistics

For all statistical analyses, confirm that the following items are present in the figure legend, table legend, main text, or Methods section.

n/a	Confirmed	
☐	☒	The exact sample size (n) for each experimental group/condition, given as a discrete number and unit of measurement
☐	☒	A statement on whether measurements were taken from distinct samples or whether the same sample was measured repeatedly
☐	☒	The statistical test(s) used AND whether they are one- or two-sided<br>*Only common tests should be described solely by name; describe more complex techniques in the Methods section.*
☐	☒	A description of all covariates tested
☐	☒	A description of any assumptions or corrections, such as tests of normality and adjustment for multiple comparisons
☐	☒	A full description of the statistical parameters including central tendency (e.g. means) or other basic estimates (e.g. regression coefficient) AND variation (e.g. standard deviation) or associated estimates of uncertainty (e.g. confidence intervals)
☐	☒	For null hypothesis testing, the test statistic (e.g. F, t, r) with confidence intervals, effect sizes, degrees of freedom and P value noted<br>*Give P values as exact values whenever suitable.*
☒	☐	For Bayesian analysis, information on the choice of priors and Markov chain Monte Carlo settings
☒	☐	For hierarchical and complex designs, identification of the appropriate level for tests and full reporting of outcomes
☒	☐	Estimates of effect sizes (e.g. Cohen's d, Pearson's r), indicating how they were calculated

Our web collection on statistics for biologists contains articles on many of the points above.

Software and code

Policy information about availability of computer code

Data collection

Bulk RNA-seq data collection: Total RNA was isolated from all tissue samples at the same time for each designated experiment. Illumina TruSeq Stranded mRNA library preparation kit was used to prepare sequencing libraries. Pooled and indexed libraries were denatured, diluted, and sequenced in single-end modus on an Illumina NextSeq 550 (Illumina Inc., San Diego, USA).

16S rRNA amplicon sequencing data collection: Genomic DNA was isolated from tissue samples at the same time for each experiment. A dual-index sequencing strategy was utilized to amplify amplicons for the hypervariable V4 region of the bacterial 16S rRNA gene (PMID: 23793624). Pooled and indexed libraries were denatured, diluted, and sequenced in paired-end modus on an Illumina MiSeq (Illumina Inc., San Diego, USA).

Single-cell mass cytometry data collection: Whole blood samples were collected via terminal bleeding. Blood was stabilized in Cytodelics stabilizer and stored at -80C until ready for analysis. Blood samples were barcoded for multiplexing using Fluidigm Cell-ID 20-Plex Pd Barcoding Kit. Pooled samples were normalized using Fluidigm EQ Four Element Calibration Beads and events were acquired on a Helios CyTOF2 mass cytometer with a wide bore configuration, using noise reduction, event length limits of 10-150 pushes and a sigma value of 3. Cells were acquired at a flow rate of 0.045ml/min.

Data analysis

Bulk RNA-seq data analysis: RNA-seq data for mus musculus were aligned using Kallisto version 0.43.1 to EnsemblDB version 79 for mRNA (genome build GRCm38.p6). For data preparation, normalization, visualization, and analysis the following open source R and Bioconductor packages were used: Bioconductor (v. 4.1), gplots (v. 3.04.0), tidyverse (v. 1.3.0), edgeR (v. 3.14.0), gt (v. 0.10), DT (v. 0.17.0), limma (v. 3.28.14), GSEABase (v. 1.34.0), Biobase (v. 2.32.0), GSVA (v. 1.12.0), gprofiler2 (0.2.0), clusterProfiler (v. 3.0.4), msigdbr (v. 7.2.1), and enrichplot (v. 1.10.2), tximport (3.5), speciateIT (v1),

16S rRNA amplicon sequencing data analysis: The sequences were demultiplexed using the dual-barcode strategy, a mapping file linking barcode to samples and split_libraries.py, a QIIME-dependent script. The resulting forward and reverse fastq files were split by sample using

the QIIME-dependent script split_sequence_file_on_sample_ids.py, and primer sequences were removed using TagCleaner (version 0.16). Further processing followed the DADA2 workflow for Big Data and DADA2 (v.1.5.2) (https://benjjneb.github.io/dada2/bigdata.html). Bacterial community matrices were built from Amplicon Sequence Variants (ASV) based on SILVA v.132 and speciateIT (http://www.speciateIT.sourceforge.net). databases. Data filtering was set to include features where 20% of its values contain a minimum of 4 counts. In addition, features that exhibit low variance across treatment conditions are unlikely to be associated with treatment conditions, and therefore variance was measured by inter-quartile range and removed at 10%. Data was normalized by cumulative sum scaling and differential abundance analysis was conducted using Linear Discriminant Analysis effect size with an FDR cut-off at q < 0.05.

Single-cell mass cytometry data analysis: All FCS files were preprocessed using Gaussian discrimination parameters, following recommendations by Fluidigm. Following this preprocessing step, FCS files were uploaded to the Astrolabe Cytometry Platform (Astrolabe Diagnostics, Inc.) where transformation, debarcoding, cleaning, labeling, and unsupervised clustering was done. Data was transformed using arcsinh with a cofactor of 5. Experimental batches were debarcoded and individual samples were then labeled using the Ek'Balam algorithm, a hierarchy-based algorithm for labeling cell subsets which combines knowledge-based gating strategy with unsupervised clustering using the FlowSOM algorithm. Differential expression analysis to compare treatment effects was conducted within the Astrolabe Cytometry Platform. Groups average t-SNE maps and unsupervised clustering using FlowSOM (v2.0.0) were generated in Cytobank.

For all other biological samples, graphs and statistical analyses were made using RStudio version 1.2.5001 and Prism version 9.1. Figures were prepared in Adobe Illustrator version 25.2.1. No custom algorithms or codes were generated or utilized in this manuscript.

For manuscripts utilizing custom algorithms or software that are central to the research but not yet described in published literature, software must be made available to editors and reviewers. We strongly encourage code deposition in a community repository (e.g. GitHub). See the Nature Research guidelines for submitting code & software for further information.

Data

Policy information about availability of data

All manuscripts must include a data availability statement. This statement should provide the following information, where applicable:

- Accession codes, unique identifiers, or web links for publicly available datasets
- A list of figures that have associated raw data
- A description of any restrictions on data availability

The RNAseq and microbiota data generated in this study have been deposited in the NCBI SRA database under accession code PRJNA768872. All other raw data are available upon request without restrictions.

Field-specific reporting

Please select the one below that is the best fit for your research. If you are not sure, read the appropriate sections before making your selection.

[x] Life sciences [ ] Behavioural & social sciences [ ] Ecological, evolutionary & environmental sciences

For a reference copy of the document with all sections, see nature.com/documents/nr-reporting-summary-flat.pdf

Life sciences study design

All studies must disclose on these points even when the disclosure is negative.

Sample size	No direct sample size calculation was conducted. Sample size estimation was conducted based on experience and published data on required number of mice per group for growth and metabolic parameters (PMID: 18768700), microbial manipulation by C-section in SPF mice (PMID: 29988069), and necessary number of replicates to increase power and accuracy in differential expression RNA-seq studies (PMID: 24319002).
Data exclusions	Routine screens for outliers were conducted for all data using Grubbs' test and robust regression followed by outlier identification. For maternal diet experiments, a prior inclusion criteria was defined as failure to gain 20% of starting body weight during the first six week of consuming the HFt-LFb diet and a peak glucose concentration of 300 mg/dL during a glucose tolerance test. Using this exclusion criteria, ~15% of females were excluded from these experiment as they showed resistance to induction of metabolic syndrome following chronic consumption of a high-fat low-fiber diet. No other exlusions were made in these experiments.
Replication	The C-section and postnatal inoculations studies were replicated in two independent studies. Studies focused on the impact of maternal diet and dysbiosis were a priori designed using a discovery and replication strategy, resulting in two independent studies. All replications were successful.
Randomization	All samples were randomized where appropriate and always for data collection, processing and analysis.
Blinding	In all studies, investigators were blinded to group identification during data collection and processing. Experimenter PJK tattooed offspring for identification while EMH, EJ, TG, LMF, and KR remained blinded to offspring treatment during offspring assessment, growth measurements, body weight measurements, metabolic readouts, and tissue collections across all experiments. Subsequently, KEM collated and analyzed offspring measures, including body weight, growth, body length, and metabolic measures. For bioinformatic analyses, samples were identified prior to pipeline initiation.

Reporting for specific materials, systems and methods

We require information from authors about some types of materials, experimental systems and methods used in many studies. Here, indicate whether each material, system or method listed is relevant to your study. If you are not sure if a list item applies to your research, read the appropriate section before selecting a response.

Materials & experimental systems

n/a	Involved in the study
☐	☒ Antibodies
☒	☐ Eukaryotic cell lines
☒	☐ Palaeontology and archaeology
☐	☒ Animals and other organisms
☐	☒ Human research participants
☒	☐ Clinical data
☒	☐ Dual use research of concern

Methods

n/a	Involved in the study
☒	☐ ChIP-seq
☒	☐ Flow cytometry
☒	☐ MRI-based neuroimaging

Antibodies

Antibodies used

Antibodies are listed using the following convention:

All antibodies were used a 1:100 dilution, with 1 µl per antibody used for up to 3 X 10^6 live cells in total staining volume of 100 µl.

Antibody (clone) - metal tag if appropriate, Company Catalog number.
CD45 (30-F11) – 89Y, Fluidigm Cat. # 3089005B;
Ly6G (1A8) – 141Pr, Fluidigm Cat. # 3141008B;
CD11c (N418) – 142Nd, Fluidigm Cat. # 3142003B;
TCRb (H57-597) – 143Nd, Fluidigm Cat. # 3143010B;
CD115 (AFS98) – 144Nd, Fluidigm Cat. # 3144012B;
CD4 (RM4-5) – 145Nd, Fluidigm Cat. # 3145002B;
CD11b (M1/70) – 148Nd, Fluidigm Cat. # 3148003B;
CD19 (6D5) – 149Sm, Fluidigm Cat. # 3149002B;
IgD (11-26c.2a) – 150Nd, Fluidigm Cat. # 3150011B;
IgM (RMM-1) – 151Eu, Fluidigm Cat. # 3151006B;
CD3 (145-2C11) – 152Sm, Fluidigm Cat. # 3152004B;
CD274/PD-L1 (10F.9G2) – 153Eu, Fluidigm Cat. # 3153016B;
CD27 (LG.3A10) – 154Sm, Fluidigm Cat. # 3150017B;
CD64 (X54-5/7.1) – 155Gd, Fluidigm Cat. # 3151012B;
CD14 (Sa14-2) – 156Gd, Fluidigm Cat. # 3156009B;
FoxP3 (FJK-16s) – 158Gd, Fluidigm Cat. # 3158003A;
F4/80 (BM8) – 159Tb, Fluidigm Cat. # 3159009B;
CD62L L-selectin (MEL-14) – 160Gd, Fluidigm Cat. # 3160008B;
CD90 (T24/31) – 161Dy, Fluidigm Cat. # 3161009B;
Ly6C (Hk1.4) – 162Dy, Fluidigm Cat. # 3162014B;
APC (APC003) – 163Dy, Fluidigm Cat. # 3163001B;
APC anti-mouse MerTK (2B10C42), BioLegend Cat. # 151508;
CX3CR1 (SA011F11) – 164Dy, Fluidigm Cat. # 3164023B;
Biotin – 165Ho, Fluidigm Cat. # 3165012B;
Biotin anti-mouse CD192/CCR2 (SA203G11), BioLegend Cat. # 150628;
CD117 cKit (2B8) – 166Er, Fluidigm Cat. # 3166004B;
CD8a (53-6.7) – 168Er, Fluidigm Cat. # 3168003B;
Ly6A/E (D7) – 169Tm, Fluidigm Cat. # 3169015B;
CD49b (HMa2) – 170Er, Fluidigm Cat. # 3170008B;
CD44 (IM7) – 171Yb, Fluidigm Cat. # 3171003B;
Maxpar Ready CD24 (M1/69), BioLegend Cat. # 101829;
Maxpar Ready NK-1.1 (PK136), BioLegend Cat. # 108743;
Maxpar Ready CD335/NKp46 (29A1.4), BioLegend Cat. # 137625;
CD45R/B220 (RA3-6B2) – 175Lu, Fluidigm Cat. # 3176002B;
FceR1a (MAR-1) – 176Yb, Fluidigm Cat. # 3176006B;
I-A/I-E (M5/114.15.2) – 209Bi, Fluidigm Cat. #3209006B.

Validation

All antibodies used in this study are commercially available and validated by manufacturers. From the manufacturer regarding technical validation: The suggested use is 1 µl for up to 3 X 10^6 live cells in 100 µl. Each lot of conjugated antibody is quality control tested by CyTOF® analysis of stained cells using the appropriate positive and negative cell staining and/or activation controls.

Animals and other organisms

Policy information about studies involving animals; ARRIVE guidelines recommended for reporting animal research

Laboratory animals	129S1/SvImJ females were used as donors for C-section experiments. C57Bl/6J females mice were used for all other experiments. C57BL/6J male mice were used breeder males. All mice were purchased from JAX at postnatal day 21.<br><br>Once at the University of Maryland, mice were housed under a 12 h light/day photoperiod with lights on an 0700 EST and ad libitum access to water and a grain-based chow diet (Purina Rodent Chow, St. Louis, MO; 28.1% protein, 59.8% carbohydrate, 12.1% fat). Timed pregnancies were established by introducing a breeder male to a cage housing two females within one hour prior to lights off. Males were removed and copulation plugs were checked within one hour of "lights on" to estimate the number of animals that were going to be used in the study. Noon on the day that the plug was observed was considered embryonic day 0.5 (E0.5).<br><br>A pre-pregnancy diet-induced obesity approach was used to determine how maternal exposures influence offspring response to the colonizing microbiome at birth. A separate cohort of postnatal day 28 C57Bl/6J females purchased from JAX were allowed to acclimate for two weeks prior to investigation. During this acclimation period, all females consumed a Low Fat – High Fiber (LFt-HFb) diet (grain-based chow, Purina 5001). Following acclimation, females were randomly assigned to the High Fat – Low Fiber diet (HFt-LFb; ResearchDiets Inc, D12492) or remained on the Low Fat – High Fiber (LFt-HFb) diet (grain-based chow, Purina 5001). Following acclimation, females were randomly assigned to the High Fat – Low Fiber diet (HFt-LFb; ResearchDiets Inc, D12492) or remained on the Low Fat – High Fiber (LFt-HFb) diet (grain-based chow, Purina 5001). Females had ad libitum access to respective diets for the span of the experiment.
Wild animals	No wild animals were used in this study.
Field-collected samples	No field-collected samples were used in this study.
Ethics oversight	University of Maryland Institutional Animal Care and Use Committee (Protocol #0517001)

Note that full information on the approval of the study protocol must also be provided in the manuscript.

Human research participants

Policy information about studies involving human research participants

Population characteristics	Deidentified human vaginal secretions were selected from a collection of vaginal swabs self-collected weekly until delivery by pregnant women enrolled in the Birth, Eating, and the Microbiome (BEAM) study at the University of Maryland Baltimore. Written informed consent was appropriately obtained from all participants. Samples were self-collected using the Copan ESwab system and stored frozen at -20°C in 1ml of Amies Transport Medium to preserve vaginal microbiota composition until transport to the laboratory where the samples were stored at -80°C.
Recruitment	As detailed in Methods, the following criteria were used for sample selection: 1) harboring a community state type (CST) I or CST IV vaginal microbiota, 2) availability of samples between 36-39 weeks of pregnancy, and 3) women remained in the respective CST throughout pregnancy.
Ethics oversight	The clinical study protocol (HP-00056389) was approved by the Institutional Review Board of the University of Maryland Baltimore.

Note that full information on the approval of the study protocol must also be provided in the manuscript.
